# Development and validation of a risk prediction model for early diabetic peripheral neuropathy based on a systematic review and meta-analysis

**DOI:** 10.3389/fpubh.2023.1128069

**Published:** 2023-02-22

**Authors:** Xixi Liu, Dong Chen, Hongmin Fu, Xinbang Liu, Qiumei Zhang, Jingyun Zhang, Min Ding, Juanjuan Wen, Bai Chang

**Affiliations:** ^1^NHC Key Laboratory of Hormones and Development, Tianjin Key Laboratory of Metabolic Diseases, Chu Hsien-I Memorial Hospital & Tianjin Institute of Endocrinology, Tianjin Medical University, Tianjin, China; ^2^Beijing Hospital of Traditional Chinese Medicine, Capital Medical University, Beijing, China

**Keywords:** type 2 diabetes mellitus, diabetic peripheral neuropathy, risk factors, prediction model, cohort study, meta-analysis

## Abstract

**Background:**

Early identification and intervention of diabetic peripheral neuropathy is beneficial to improve clinical outcome.

**Objective:**

To establish a risk prediction model for diabetic peripheral neuropathy (DPN) in patients with type 2 diabetes mellitus (T2DM).

**Methods:**

The derivation cohort was from a meta-analysis. Risk factors and the corresponding risk ratio (RR) were extracted. Only risk factors with statistical significance were included in the model and were scored by their weightings. An external cohort were used to validate this model. The outcome was the occurrence of DPN.

**Results:**

A total of 95,604 patients with T2DM from 18 cohorts were included. Age, smoking, body mass index, duration of diabetes, hemoglobin A1c, low HDL-c, high triglyceride, hypertension, diabetic retinopathy, diabetic kidney disease, and cardiovascular disease were enrolled in the final model. The highest score was 52.0. The median follow-up of validation cohort was 4.29 years. The optimal cut-off point was 17.0, with a sensitivity of 0.846 and a specificity of 0.668, respectively. According to the total scores, patients from the validation cohort were divided into low-, moderate-, high- and very high-risk groups. The risk of developing DPN was significantly increased in moderate- (RR 3.3, 95% CI 1.5–7.2, *P* = 0.020), high- (RR 15.5, 95% CI 7.6–31.6, *P* < 0.001), and very high-risk groups (RR 45.0, 95% CI 20.5–98.8, *P* < 0.001) compared with the low-risk group.

**Conclusion:**

A risk prediction model for DPN including 11 common clinical indicators were established. It is a simple and reliable tool for early prevention and intervention of DPN in patients with T2DM.

## 1. Introduction

The number of people with diabetes mellitus (DM) and its comorbidities is growing rapidly worldwide. Diabetic peripheral neuropathy (DPN) is a major complication of diabetes, and at least 50% of patients with diabetes will develop DPN in their lifetime (1, 2). There is a large amount of evidence revealing that DPN is an important factor in the development of diabetic foot ulcer, Charcot neuroarthropathy (3), and even non-traumatic lower-limb amputation (1, 4). Besides, dysfunction of small and large fibers leads to abnormal foot temperature, pain sensation, and proprioception, which ultimately result in repeated foot damage and imbalance, increasing the risk of falls and fractures (5, 6). Furthermore, DPN is a predictor of mortality in patients with diabetes. A 13-year prospective study of patients with diabetes showed that DPN was significantly associated with cardiovascular and all-cause mortality (7). Considering the devastating consequences, DPN has been a public health problem that pose a significant challenge to social, financial and health care systems (8, 9). Unfortunately, there is currently a lack of early diagnosis and effective clinical intervention. DPN is usually insidious and is missed in the onset until it is well-established, at the point it seems to be irreversible (10). So early prevention is critical to tackle this issue.

Hyperglycemia has been recognized as the most important risk factor for DPN in patients with type 2 diabetes mellitus (T2DM). However, the UK prospective diabetes study (UKPDS) (11), the intensified multifactorial intervention in patients with type 2 diabetes (steno-2) study (12) and other large clinical intervention trials (13–15) did not find the benefit of glucose control on the occurrence and development of DPN in patients with T2DM. Antidiabetic treatment alone is insufficient to prevent DPN in individuals with T2DM (16). Besides hyperglycemia, T2DM coexists with other metabolic disorders, such as obesity, dyslipidemia, and hypertension, etc. Recent evidence suggests that these multiple metabolic disorders are involved in DPN onset. Early recognition and comprehensive assessment of these related risk factors allow for an earlier identification of high-risk individuals and an earlier management of DPN. Therefore, a risk prediction model for DPN including related risk factors is developed in this study, and it may be a more effective strategy for preventing DPN.

## 2. Methods

### 2.1. Study registration

The protocol was registered in the International Prospective Register of Systematic Reviews (PROSPERO) with the registration number CRD42021246320.

### 2.2. Study populations

#### 2.2.1. Derivation cohort

**Figure 1:**
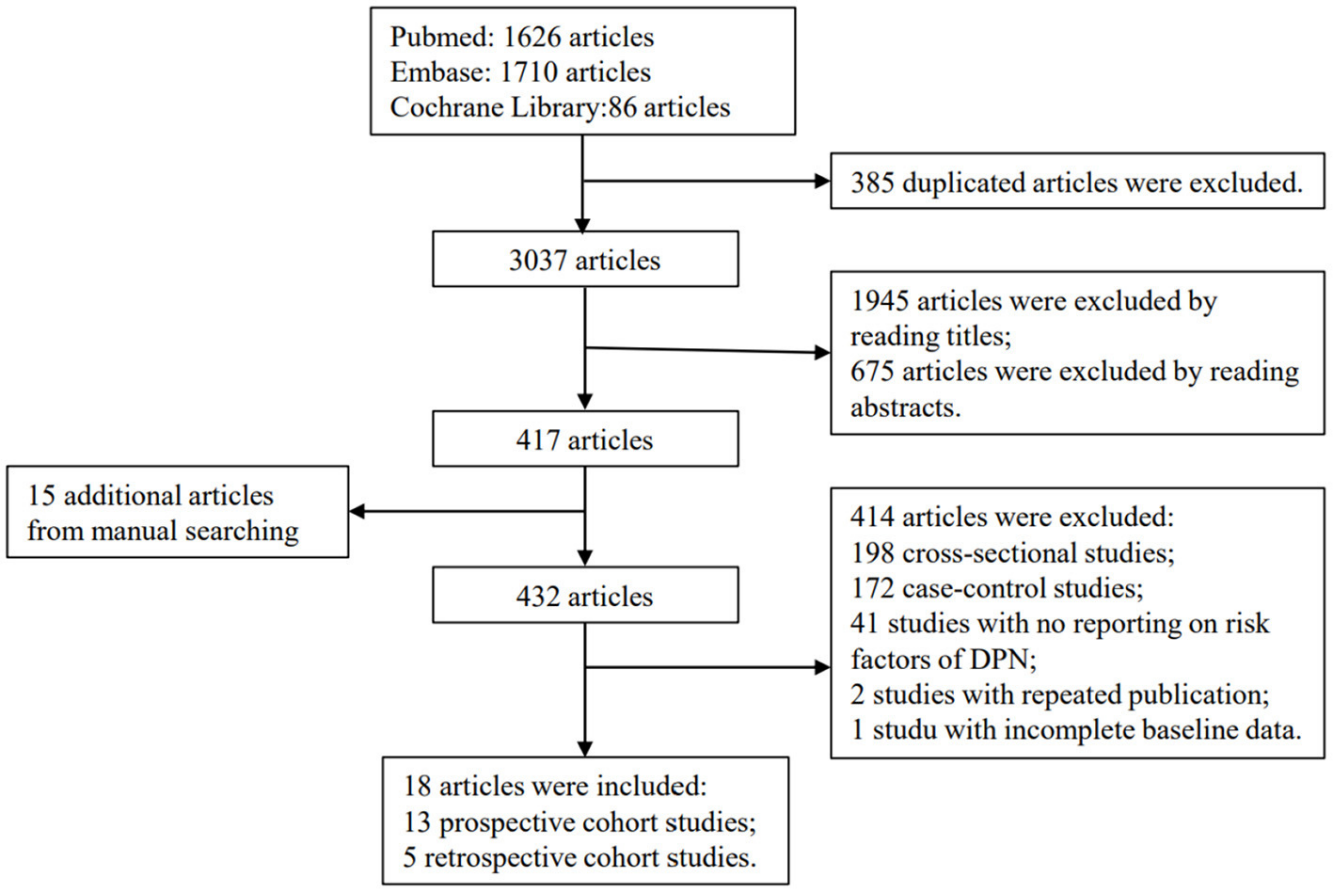
Flow diagram of literature selection process.

The derivation cohort patients came from a systematic review and meta-analysis. We searched the electronic databases including Pubmed, Embase, and Cochrane Library from inception to May 2020, using the following medical subject heading terms and their keywords: “diabetes mellitus, type 2,” “diabetic neuropathies,” “risk factors,” and “cohort studies.” Ultimately, a total of 95,604 patients with T2DM from 13 prospective cohorts and 5 retrospective cohorts were included. The research subjects were mainly from 19 countries and regions, of which 50% were from Asia, 22.22% were from Europe, 22.22% were from America, and 5.56% were from Oceania. All the 18 cohort studies reported the risk ratio (RR) with 95% confidence interval (CI) of each risk factor, and they were of high quality as assessed by the Newcastle-Ottawa Scale (NOS; provided in Supplementary Table 2). A flow diagram of literature selection process is shown in Figure 1. Details of literature search strategy, inclusion and exclusion criteria, data extraction, publication bias, and quality assessment are shown in Supplementary material.

#### 2.2.2. Validation cohort

**Figure 2:**
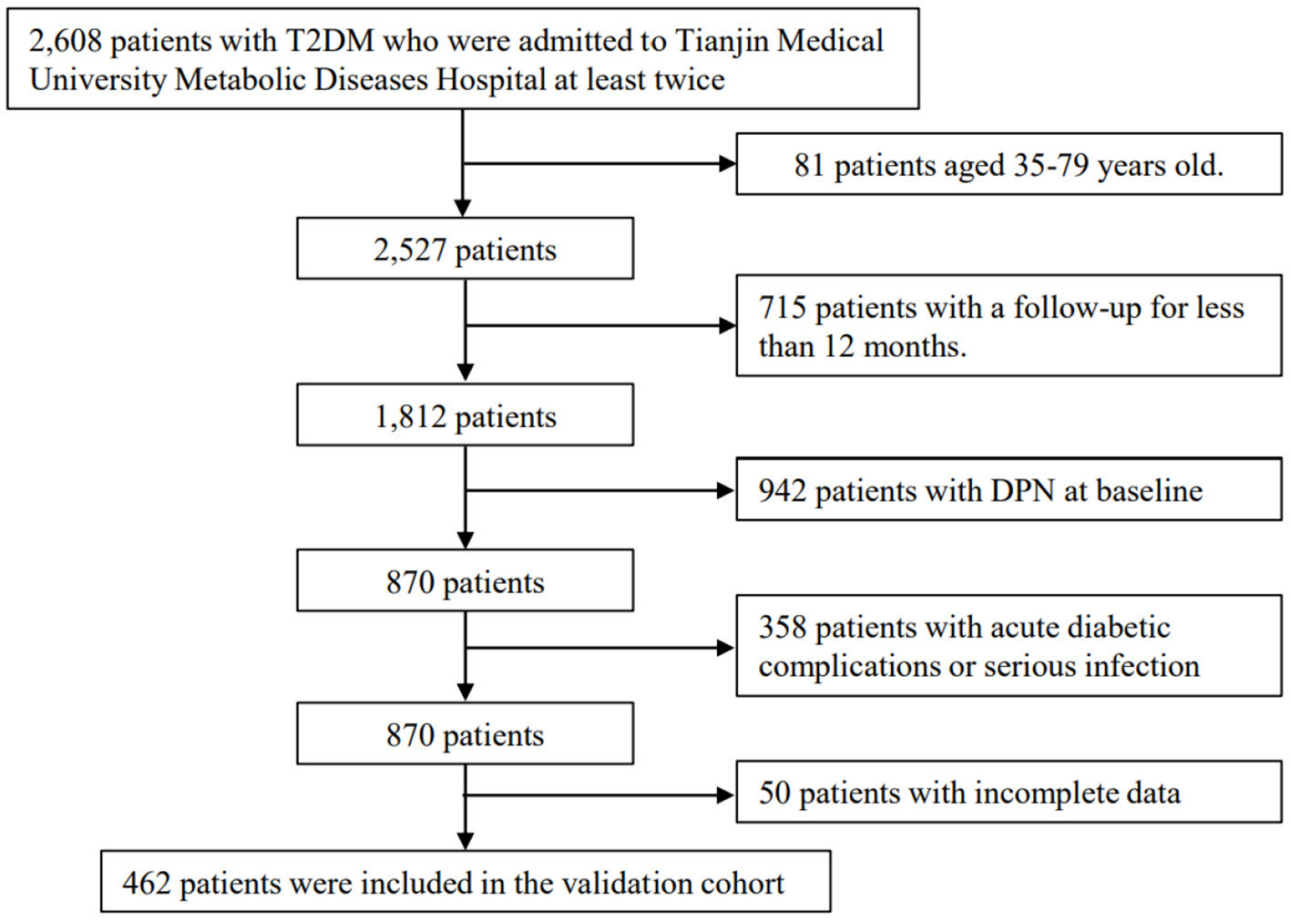
Process for the selection of patients in the validation cohort.

In total, 2,608 patients with T2DM who were admitted to Tianjin Medical University Metabolic Diseases Hospital at least twice (baseline from September 2010 to September 2020) were considered for our study. We further selected patients aged 35–79 years old, without DPN at baseline, and with a follow-up of more than 12 months for inclusion in the validation cohort. Exclusion criteria included the presence of acute complications, serious infection, myocardial infarction, stroke, and cancer. We excluded 81 patients aged <35 or >79 years old, 715 patients with a follow-up for <12 months, 942 patients with DPN at baseline, 358 patients with acute diabetic complications or serious infection, and 50 patients with incomplete data. Finally, 462 patients were selected as the retrospective validation cohort. The flowchart is shown in Figure 2.

### 2.3. Outcome

The outcome was the occurrence of DPN. DPN was diagnosed by a combination of symptoms, signs and nerve conduction function consistent with the guideline provided by the 2010 Toronto Consensus (17).

### 2.4. Definitions

Smoking was defined as a total number of ≥100 cigarettes in their lifetime (18). Low high-density lipoprotein (HDL-c) was defined as HDL-c <1.3 mmol/L. High triglyceride (HTG) was defined as TG ≥ 1.7 mmol/L. Hypertension (HTN) was defined as systolic blood pressure (SBP) ≥ 140 mmHg and/or diastolic blood pressure (DBP) ≥ 90 mmHg. Diabetic retinopathy (DR) was confirmed by ophthalmoscopy. Diabetic kidney disease (DKD) was identified clinically by an estimated glomerular filtration rate (eGFR) <60 mL/min/1.73 m^2^ and/or urinary albumin-to-creatinine ratio (UACR) ≥ 30 mg/g caused by diabetes mellitus for ≥3 months (19). Cardiovascular diseases (CVD) included angina, previous myocardial infarction, or electrocardiographic manifestations of coronary ischemia.

## 3. Statistical analysis

### 3.1. Meta-analysis

The RR value and 95% CI of each risk factor were extracted from the included cohorts, and then pooled to screen out risk factors according to the heterogeneity across studies. Heterogeneity test was analyzed by *Q*-test, and measured by *I*^2^-value. When there was statistically significant heterogeneity (*P*-value < 0.10 or *I*^2^-value > 50%), the pooled RR and 95% CI were generated by a random effects model, otherwise by a fixed effects model. Subgroup analyses were performed according to the magnitude of the increase in continuous variables. Continuous variables included age (years, increment by 1 vs. 5–10), BMI (kg/m^2^, increment by 1–5), and duration of DM (years, increment by 1 vs. 5–10). Sensitivity analyses were conducted to evaluate the robustness of the results after a single study was omitted. Publication biases were determined using Begg's and Egger's linear regression tests, and the latter one prevailed if the two results were inconsistent. All tests were considered statistically significant at two-tailed *P*-value < 0.05, except for heterogeneity test and publication bias were at *P*-value < 0.1. Statistical analyses were performed with Stata software (version 12.0 StataCorp, College Station, TX).

### 3.2. Model development

We developed a risk score system, which is simple and convenient for clinical practice. First, all the risk factors with appropriate stratifications from the above systematic review and meta-analysis were incorporated into the model. We selected appropriate RR value and 95% CI to calculate the corresponding β-coefficient (β-coefficient), which represents the multiple increase in the risk of an individual developing a certain disease when each risk variable increases by one level. Second, multiplying β-coefficient by 10, and then rounding it to one decimal place (20), we further obtained the respective score of each risk factor. At last, all risk factors were stratified and assigned scores to construct a risk prediction model for DPN according to meta-analysis and clinical practice guidelines. The total score was calculated by adding up the score of each risk factor (21). For individuals, the higher the cumulative score, the higher risk of DPN in the future.

### 3.3. Model validation

Continuous variables with normal distributions were expressed as mean ± standard deviation, and those with skewed distributions were described as median (interquartile range). Categorical variables were represented by frequency (percentage). A receiver operating characteristic (ROC) curve was performed based on the total scores. The sensitivity, specificity, the area under ROC curve (AUC) values, and optimal cut-off point were calculated. The AUC means prediction accuracy, with the value ranging from 0.5 to 1.0. The higher the AUC value, the better the prediction accuracy. The optimal cut-off point with higher sensitivity and a certain specificity was determined according to the Youden index. According to the optimal cumulative score, patients were segmented into four risk groups, including low-, moderate-, high-, and very high-risk. Kaplan–Meier curves were conducted to evaluate the cumulative risk of morbidity in different groups. Statistical analyses were performed using the SPSS 26.0 (IBMCorp, Armonk, NY, USA) and Stata software version 12.0 (StataCorp, College Station, TX).

## 4. Results

### 4.1. Description of the cohorts

#### 4.1.1. Derivation cohort

We roughly analyzed the baseline data of participants from the included cohorts. A total of 95,604 patients with T2DM were included in the derivation cohort, with age between 35 and 79 years old, male accounting for 49.6%, and duration of DM ranging 1–19 years. The follow-up was 1–13 years, equivalent to 95,604 to 1,242,853 person-years. Among the patients, the mean body mass index (BMI) ranged 24.9–31.0 kg/m^2^, mean hemoglobin A1c (HbA1c) ranged 7.0–8.7% (53.0–71.6 mmol/mol), mean SBP ranged 135–143 mmHg, mean DBP ranged 77–87 mmHg, mean HDL-c ranged 1.30–3.40 mmol/L, and mean TG ranged 1.37–9.91 mmol/L. 6.6–80.2% of participants were smokers. 23.4–88.1% were with hyperlipidemia, 41.5–77.8% were with hypertension, 12.0–32.1% were with DR, 14.6–43.4% were with DKD, and 6.0–44.6% were with CVD. Across the studies, 54.2–88.9% received oral antidiabetic drugs (OAD), 1.0–47.8% received insulin injection, 7.8–80.0% received stains, and 18.5–82.8% received angiotensin converting enzyme inhibitors (ACEI) or angiotensin receptor blockers (ARB). During follow-up, 19,399 DPN events were observed, with an estimated incidence of 20.3%. There were 24 risk factors available from these studies, including age, gender, marital status, smoking, height, BMI, waist circumference, duration of DM, fasting plasma glucose (FPG), HbA1c, total cholesterol (TC), TG, HDL-c, low density lipoprotein (LDL-c), SBP, DBP, C-reactive protein, eGFR, hypertension, DR, DKD, CVD, insulin, and statins. Baseline characteristics and risk factors of the 18 cohorts are provided in Supplementary Tables 1, 3.

#### 4.1.2. Validation cohort

A total of 462 patients with T2DM were enrolled, including 315 males (68.2%). The median follow-up time was 4.29 years, and 249 patients (162 males and 87 females) developed DPN at the end of follow-up. The incidence was 53.8%. Among all patients at baseline, the mean age was 52.4 ± 12.2 years old, duration of DM was 6.0 (3.0–11.0) years, mean BMI was 27.49 ± 4.43 kg/m^2^, mean HbA1c was 8.52 ± 1.88% (69.5 ± 20.5 mmol/mol), mean SBP was 136 ± 66 mmHg, mean DBP was 81 ± 11 mmHg, mean HDL-c was 1.19 ± 0.28 mmol/L, and TG was 1.63 (1.15, 2.56) mmol/L. Two hundred (43.3%) participants were smokers. Two hundred and thirty-six (51.1%) had hypertension, 109 (23.6%) had DR, 120 (26.0%) had DKD, and 224 (48.5%) had CVD. Four hundred and twenty-seven (92.4%) patients received OAD, 269 (58.2%) received insulin, 183 (39.6%) received statins, and 186 (40.3%) received ACEI or ARB. Baseline data of the validation cohort are shown in Supplementary Table 5.

### 4.2. Model development

**Figure 3:**
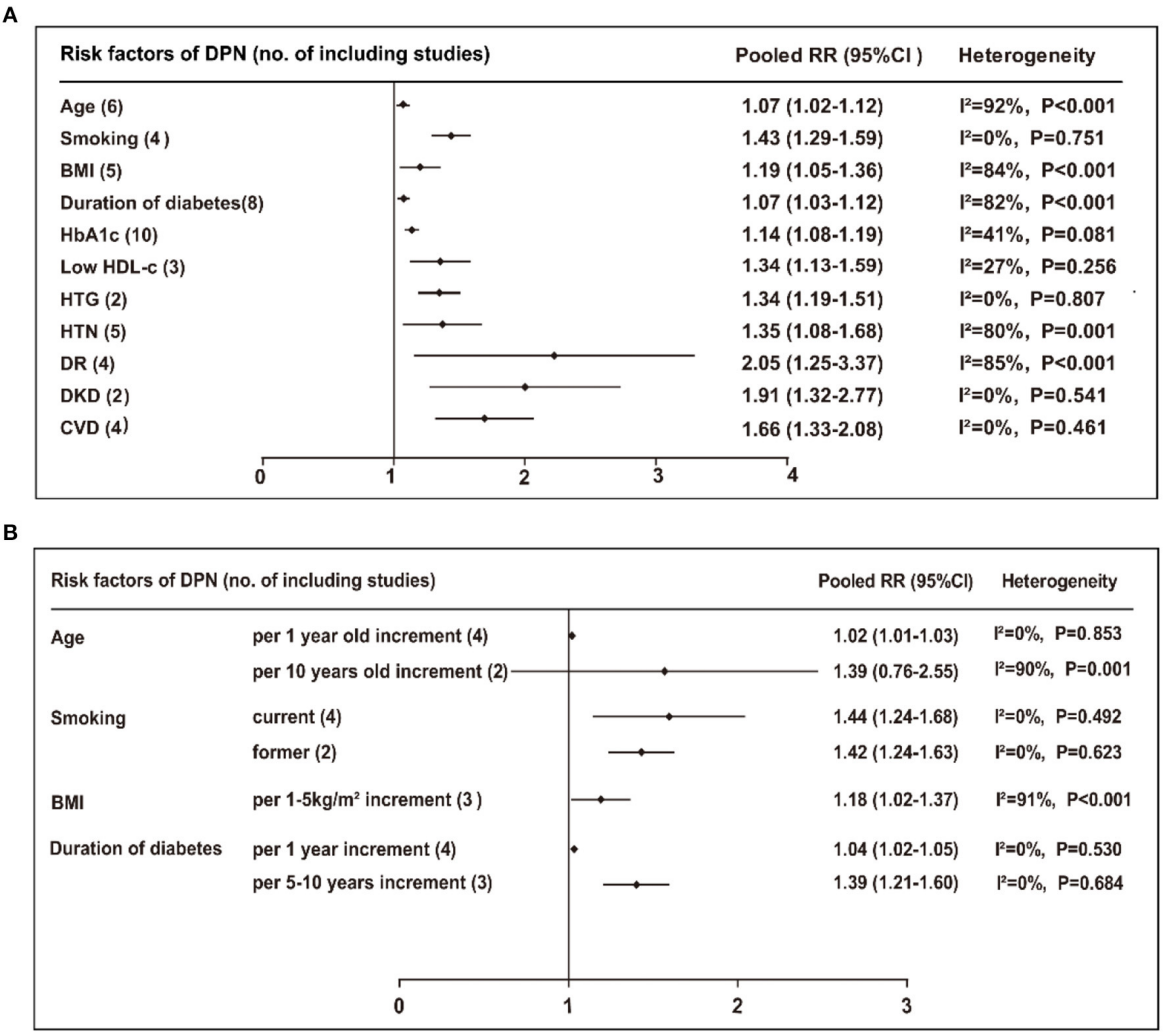
**(A)** Pooled RR (95% CI) and heterogeneity test of the risk factors for developing DPN. **(B)** Subgroup or sensitivity analyses of the risk factors for DPN. BMI, body mass index; HbA1c, Hemoglobin A1c; HDL-c, high density lipoprotein cholesterol; HTG, high triglyceride; HTN hypertension; DR, diabetic retinopathy; DKD, diabetic kidney disease; CVD, cardiovascular disease.

Of the 24 risk factors identified from the above meta-analysis, 11 risk factors were involved in DPN onset. The risk stratification methods were carefully selected by subgroup or sensitivity analyses, which were most reasonable considering the feasibility and convenience of clinical practice. These 11 risk factors included in the final model were as follows: age incremented by 1 year (RR 1.02, 95% CI 1.01–1.03, *P* = 0.001; β-coefficient 0.020, score 0.2), smoking (RR 1.43, 95% CI 1.29–1.59, *P* < 0.001; β-coefficient 0.358, score 3.0), BMI incremented by 1–5 kg/m^2^ (RR 1.18, 95% CI 1.02–1.37, *P* = 0.030; β-coefficient 0.166, score 1.5), duration of diabetes incremented by 5–10 years (RR 1.39, 95% CI 1.21–1.60, *P* < 0.001; β-coefficient 0.329, score 3.0), HbA1c incremented by 1% (RR 1.14, 95% CI 1.08–1.19, *P* < 0.001; β-coefficient 0.131, score 1.5), low HDL-c (RR 1.34, 95% CI 1.13–1.59, *P* = 0.001; β-coefficient 0.293, score 3.0), high triglyceride (HTG; RR 1.34, 95% CI 1.19–1.51, *P* < 0.001; β-coefficient 0.293, score 3.0), hypertension (RR 1.35, 95% CI 1.08–1.68, *P* = 0.008; β-coefficient 0.300, score 3.0), DR (RR 2.05, 95% CI 1.25–3.37, *P* = 0.005; β-coefficient 0.718, score 7.0), DKD (RR 1.91, 95% CI 1.32–2.77, *P* = 0.001; β-coefficient 0.647, score 6.5), and CVD (RR 1.66, 95% CI 1.33–2.08, *P* < 0.001; β-coefficient 0.507, score 5.0). A forest plot of heterogeneity test of 11 risk factors is presented in Figure 3A, and subgroup and sensitivity analyses are shown in Figure 3B. These risk factors, risk stratification, RRs, 95% CIs, β-coefficients, and risk scores are shown in Supplementary Table 6.

According to the stratifications and scores of the above risk factors, a simple DPN risk prediction model was developed as follows: age (years, 35–49 = 0, 50–59 = 2.0, 60–69 = 4.0, 70–79 = 6.0), smoking (no = 0, yes = 3.5), BMI (kg/m^2^, <24.00 = 0, 24.00–27.99 = 1.5, ≥28.00 = 3.0), duration of DM (years, <5.0 = 0, 5.0–9.9 = 2.5, 10.0–19.9 = 5.0, ≥20.0 = 7.5), HbA1c (%, <7.0 = 0, 7.0–7.9 = 1.5, 8.0–8.9 = 3.0, ≥9.0 = 4.5), HDL-c (mmol/L, ≥1.30 = 0, <1.30 = 3.0), TG (mmol/L, <1.70 = 0, ≥1.70 = 3.0), hypertension (no = 0, yes = 3.0), DR (no = 0, yes = 7.0), DKD (no = 0, yes = 6.5), and CVD (no = 0, yes = 5.0; shown in Table 1).

**Table 1:** Risk prediction model for DPN^#^.

**Risk factors of DPN**	**Risk stratification**	**Score**
Age (year)^##^	35–49	0
	50–59	2.0
	60–69	4.0
	70–79	6.0
Smoking^###^	No	0
	Yes	3.5
BMI (kg/m^2^)^####^	<24.00	0
	24.00–27.99	1.5
	≥28.00	3.0
Duration of DM (year)	<5.0	0
	5.0–9.9	2.5
	10.0–19.9	5.0
	≥20.0	7.5
HbA1c (%)	<7.0	0
	7.0–7.9	1.5
	8.0–8.9	3.0
	≥9.0	4.5
HDL-c (mmol/L)	≥1.30	0
	<1.30	3.0
TG (mmol/L)	<1.70	0
	≥1.70	3.0
HTN	No	0
	Yes	3.0
DR	No	0
	Yes	7.0
DKD	No	0
	Yes	6.5
CVD^#####^	No	0
	Yes	5.0

# This model was suitable for predicting the risk of DPN in patients with type 2 diabetes, with a total score of 52.0. Those with scores ≥17.0 were considered as high-risk groups.

## Patients from the derivation and validation cohort studies aged 35–79 years old.

### Smoking was defined as the total amount of smoking ≥100 cigarettes in their lifetime.

#### It is recommended to adopt different criteria in white and Asian patients.

##### Cardiovascular diseases (CVD) included angina, previous myocardial infarction, or electrocardiographic manifestations of coronary ischemia.

### 4.3. Model validation

**Figure 4:**
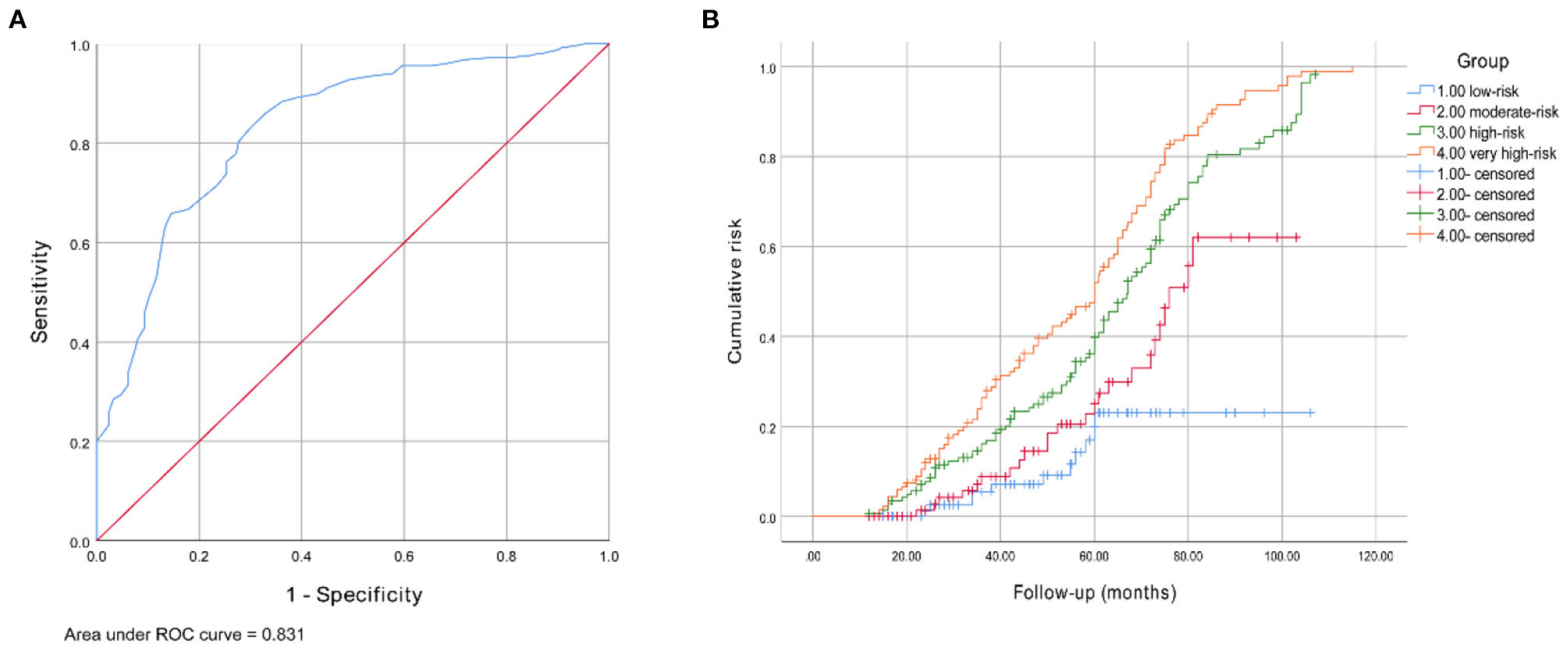
**(A)** Receiver operating characteristic curve for the DPN risk prediction model. The AUC and 95% CI were 0.831 (0.794–0.868). **(B)** Kaplan-Meier curve of DPN end point for four risk group: moderate- (RR 3.3, 95% CI 1.5–7.2, *P* = 0.020), high- (RR 15.5, 95% CI 7.6–31.6, *P* < 0.001), and very high-risk groups (RR 45.0, 95% CI 20.5–98.8, *P* < 0.001).

In the validation cohort, the AUC value of this model was 0.831 (95% CI 0.794–0.868, *P* < 0.001). The ROC curve is provided in Figure 4A. The purpose of constructing this model was to early identify the high-risk population of DPN, so the sensitivity should be as high as possible on the premise of ensuring a certain degree of specificity when selecting the cut-off point. Therefore, a score of 17.0 was finally selected as the best predictive cut-off point, with a higher sensitivity of 0.846, a specificity of 0.668, and a maximum Youden index value of 0.531. Sensitivity, specificity and Youden indexes of different cut-off scores are shown in Supplementary Table 7. Based on the frequencies of the total risk scores, 462 patients were divided into four groups: low- (*n* = 97, 0–12.5 scores), moderate- (*n* = 81, 13.0–16.5 scores), high- (*n* = 149, 17.0–24.5 scores), and very high-risk (*n* = 135, 25.0–52.0 scores), and the corresponding numbers of patients who developed DPN at the end of the follow-up were 11 (11.3%), 24 (29.6%), 99 (66.4%), and 115 (85.2%), respectively. The risk of developing DPN was significantly increased in moderate- (RR 3.3, 95% CI 1.5–7.2, *P* = 0.020), high- (RR 15.5, 95% CI 7.6–31.6, *P* < 0.001), and very high-risk groups (RR 45.0, 95% CI 20.5–98.8, *P* < 0.001) compared with the low-risk group. The Kaplan-Meier curves for these four groups are shown in Figure 4B. The cumulative risk for each group was provided in Table 2.

**Table 2:** Prevalence of DPN in four risk groups stratified by risk scores in the validation cohort.

**Risk stratification**	**No. of patients (*n* = 462)**	**No. of events (*n* = 249)**	**Prevalence rate (%)**	**RR (95% CI)**	***P***
Low	97	11	11.3		
Moderate	81	24	29.6	3.3 (1.5–7.2)	0.020
High	149	99	66.4	15.5 (7.6–31.6)	<0.001
Very high	135	115	85.2	45.0 (20.5–98.8)	<0.001

## 5. Discussion

Recent evidence suggests that multiple metabolic disorders are all involved in DPN onset, but the results of different studies were not entirely consistent. Given that high-quality meta-analysis is at the top of the evidence-based medicine level pyramid, and it helps to establish a more robust prediction model than a single study. Meta-analysis was applied to integrate relevant cohort studies (22–39). We screened out the risk factors of DPN from a meta-analysis, and constructed a simple risk prediction model for DPN by quantitatively evaluating risk factors. This model provided quantitative standards for early identifying high-risk groups, thus we can develop comprehensive and individualized prevention and intervention strategies.

We included 18 cohort studies with a total of 95,604 patients, and screened out 11 risk factors of DPN, including age, smoking, BMI, duration of DM, HbA1c, HDL-c, TG, hypertension, DR, DKD, and CVD. Except age and duration of DM were non-modifiable, the remaining factors can be modifiable by lifestyle optimization and drug intervention. We recommended that patients at low-risk improve self-monitoring and conduct regular risk assessment; High-risk groups, based on risk assessment, actively improve lifestyle, such as quitting smoking, optimizing diet structure, participating in moderate physical exercise, and weight control, etc. They should optimize the basic controlling of blood glucose, lipids and blood pressure, and further intervene in diabetic complication under the guidance of professional doctors. It should be noted that once a patient is classified as more than low risk, neurological examination for DPN should be performed, especially the sensitive tests for small fiber neuropathy, to monitor the stage and progression of DPN. Through systematic clinical prevention strategies, we can reduce the incidence of DPN, improve life quality of patients, and save costs of clinical management.

In the risk factors for DPN from our meta-analysis, DR, DKD, and CVD were the most powerful, and they usually share common factors and are often co-morbid. Our study suggested that both DR and DKD were increased the risk of DPN by nearly 2 times, and CVD increased the risk by ~1.7 times. Duration of DM, smoking, low HDL-c, high TG and hypertension were the relatively moderate risk factors. Previous cross-sectional and cohort studies shown that the incidence of DPN reached 50% after 8 years of diagnosis in diabetic patients (34). We indicated that the risk of DPN increased by 39% for every 10-year increment in the duration of DM. Smoking is an independent risk factor for DPN in diabetic patients (40). Consistent with previous researches, our study reported the risk increased by about 40%, whether quitting smoking or not. Smoking was defined as a total number of ≥ 100 cigarettes in their lifetime (18). Dyslipidemia is closely correlated with the progression of DPN, especially high TG and low HDL-c levels (34, 37). As TG ≥ 1.70 mmol/L or HDL-c <1.30 mmol/L, the risk for DPN increased by 34% in this study. We found no statistical significance when it came to LDL-c, and there is still a lack of research data. Considering that patients with T2DM are prone to comorbid dyslipidemia, the widespread use of statins at baseline may affect the results. Hypertension plays a crucial role in the occurrence and development of DPN. Our result reported that hypertension increased the risk of DPN by 35%, but no correlation was found between SBP, DBP, and DPN. The relationship between blood pressure and DPN risk still needs to be investigated in large-scale prospective studies. Age, HbA1c, and BMI were also major risk factors of DPN. Prospective researches have shown that age was independently related with DPN. For every 10 years increment in age, the risk of DPN was increased by 20% in this study. According to study population included in our meta-analysis, the results were mainly applicable to patients with T2DM aged 35–79 years old. Patients with type 1 diabetes mellitus tend to be younger, and there is currently a lack of data on DPN risk in patients with early-onset (age <35 years old) T2DM. Further prospective studies with large samples are needed. With HbA1c incremented by 1%, the risk of DPN increased by 14% in this study. We did not find a correlation between FPG and DPN. FPG is the blood glucose level at a certain time point, while HbA1c reflects the average level in the recent 2–3 months. Therefore, HbA1c could better reflect blood glucose control. BMI is the most common measure of obesity and plays a crucial role in initiation of DPN. Obesity is often associated with insulin resistance and dyslipidemia, which are components of cardiovascular disease risk factors, and their interaction will also increase the risk of DPN (41). When BMI incremented by 1–5kg/m^2^, the risk of DPN increased by 18% in our study. The difficulty lied in the excessive stratification of a certain risk factor and the occurrence of extreme value, which led to its excessive weight. In order to avoid this influence, appropriate results and stratification methods were selected for some risk factors. For example, considering the weight of BMI is too high, we converted it into categorical variable. According to Chinese standards for BMI (42), the population was divided into three groups: normal (<24.00 kg/m^2^), overweight (24.00–27.99 kg/m^2^), and obese (≥28.00 kg/m^2^). We defined BMI < 24.00 kg/m^2^ as the normal group with a 0 score, BMI ranged 24.00–27.99 kg/m^2^ as the overweight group with a score of 1.5, and BMI ≥28.00 kg/m^2^ as the obese group with a score of 3.0.

Some investigators have established prediction models for DPN (43–46). However, those models were mostly constructed based on cross-sectional studies, small sample cohort studies or *post-hoc* analysis of randomized controlled trials. A prior model developed by Basu et al. (44) used complex computer algorithms, which is not convenient for clinical promotion. Hence, based on meta-analysis from 18 cohort studies with 95,604 patients with T2DM, we established a simple and robust risk prediction model for DPN that consisting of lifestyle and clinical data, including age, smoking, BMI, duration of DM, HbA1c, low HDL-c, high TG, hypertension, DR, DKD and CVD.

Furthermore, patients with T2DM from China were included as an external cohort to verify the predictive performance of this model. AUC of our model was 0.831, indicating good predictive performance. Since this model aimed at early identification of high-risk population of DPN, the sensitivity should be improved as far as possible on the premise of ensuring a certain degree of specificity when selecting cut-off point. Finally, a score of 17.0 with a higher sensitivity of 0.846 and a specificity of 0.668 was selected as the optimal cut-off point. Sensitivity represents the true positive rate, our model achieved a high sensitivity, indicating that the authenticity of positive prediction is relatively high, which is in line with the purpose of the prediction model. Specificity stands for the true negative rate. There is a shortcoming that the true negative rate is slightly low. The reason may be that some indicators which may affect the occurrence and development of DPN were not included due to the meta-analysis, such as C-reactive protein, erythrocyte sedimentation rate, fibrinogen, and D-dimer, etc. Few related cohort researches were available in this meta-analysis, so we failed to include them in our model. Further prospective studies on these indicators are still needed, and we expect to include them to improve the model's specificity. Patients with a cumulative score of ≥17.0 were at high-risk of DPN onset. According to the total scores of participants, we further divided them into four groups, namely, low-, moderate-, high- and very high-risk groups. Compared with the low-risk group, the moderate-, high- and very high-risk groups had 3.3-, 15.5-, and 45.0-fold increases in the rate of developing DPN, respectively. Through the application of this model to assess the risk factors and make targeted measures, it is expected to transform high-risk individuals into lower-risk groups, so as to achieve dynamic management and ultimately reduce the occurrence of DPN.

Nevertheless, the study presented here faces some limitations. First, heterogeneity among literatures is inevitable because of differences in study design and diversities in race and sex compositions of the included studies. Although subgroup analysis and sensitivity analysis were further conducted to minimize heterogeneity, the causes for heterogeneity of some factors were still not explicit. Second, the number of included researches on some risk factors is small. Some other clinical indicators, such as C-reactive protein, erythrocyte sedimentation rate, fibrinogen and D-dimer, may involve in the progression of DPN, but few related cohort studies were retrievable, so we failed to include them in our model. All these confounding factors may bias the results. We expect more prospective studies on these factors to be explored and included them to update our model in the future. Third, participants in the derivation cohort were from several countries and regions, while the validation cohort was only consisted of Chinese patients. Therefore, multicenter external cohorts remained to verify the predictive performance of this model.

## 6. Conclusion

Based on meta-analysis, we developed a simple and reliable risk prediction model for DPN in combined with lifestyle and clinical data, including age, smoking, BMI, duration of DM, HbA1c, low HDL-c, high TG, hypertension, DR, DKD, and CVD. This model is important for early prevention and individual intervention of DPN.

## Data availability statement

The original contributions presented in the study are included in the article/Supplementary material, further inquiries can be directed to the corresponding author.

## Ethics statement

The studies involving human participants were reviewed and approved by the Ethics Committee of Tianjin Medical University Metabolic Diseases Hospital. Written informed consent for participation was not required for this study in accordance with the national legislation and the institutional requirements.

## Author contributions

XixL and DC conducted the research, collected the data, statistical analysis, and wrote the manuscript. HF, XinL, QZ, JZ, MD, and JW contributed to the discussion, performed study quality assessment, and reviewed the manuscript. BC contributed to concept, design, and manuscript revision. All authors contributed to the article and approved the submitted version.

## Supplementary material

Systematic review and meta-analysis to identify the risk factors of diabetic peripheral neuropathy onset in patients with type 2 diabetes mellitus is available in the Supplementary material.
