# Supplementary material for: Development and validation of a risk prediction model for early diabetic peripheral neuropathy based on a systematic review and meta-analysis
Source: Front Public Health. 2023 Feb 22;11:1128069. doi: 10.3389/fpubh.2023.1128069 (PMC9992641; doi:10.3389/fpubh.2023.1128069)
Supplement: Supplementary file 1 [file Data_Sheet_1.PDF]

Supplementary Material

Development and Validation of a Risk Prediction Model for Early Diabetic Peripheral Neuropathy Based on a Systematic Review and Meta-Analysis

Xixi Liu[1†], Dong Chen[2†], Hongmin Fu[1], Xinbang Liu[1], Qiumei Zhang[1], Jingyun Zhang[1], Min Ding[1], Juanjuan Wen[1], and Bai Chang[2*]

*** Correspondence:** Bai Chang: changbai1972@126.com

1 Systematic review and meta-analysis to identify the risk factors of diabetic peripheral neuropathy onset in patients with type 2 diabetes mellitus

This study was conducted following the Preferred Reporting Items for Systematic Review and Meta-analysis (PRISMA)(1) and the Meta-analysis of Observational Studies in Epidemiology (MOOSE)(2). All processes were completed independently by two authors. If there is any disagreement, two other authors will be consulted to attain consensus.

Literature search strategy

We searched the electronic databases including Pubmed, Embase, and the Cochrane Library from inception to May 2020, using the following medical subject heading (Mesh) terms and their keywords: "diabetes mellitus, type 2", "diabetic neuropathies", "risk factors", and "cohort studies". There was no restriction on language of publication.

Detailed search strategy in Pubmed

#1 "diabetes mellitus, type 2"[MeSH Terms] OR "type 2 diabetes mellitus"

#2 "diabetic neuropathies"[MeSH Terms] OR "somatosensory disorders"[MeSH Terms] OR "reflex, abnormal"[MeSH Terms] OR "neural conduction"[MeSH Terms] OR "diabetic neuropath*" OR "diabetic peripheral neuropath*" OR "diabetic sensorimotor polyneuropath*" OR "distal symmetrical polyneuropath*" OR "hypesthesia" OR "paresthesia" OR "hyperalgesia" OR "hyperesthesia" OR "numbness" OR "tingling" OR "unsteadiness" OR "prickling" OR "burning pain" OR "ankle reflex" OR "achilles reflex" OR "hyporeflexia" OR "touch sensation" OR "touch perception" OR "temperature sensation" OR "temperature perception" OR "vibration sensation" OR "vibration perception" OR "Rydel-Seiffer fork" OR "pinprick sensation" OR "pinprick perception" OR "10-g monofilament test" OR "Semmes-Weinstein" OR "nerve conduction"

#3 "risk factors"[MeSH Terms] OR "hyperglycemia"[MeSH Terms] OR "glycated hemoglobin A"[MeSH Terms] OR "hyperlipidemias"[MeSH Terms] OR "triglycerides"[MeSH Terms] OR "lipids"[MeSH Terms] OR "cholesterol"[MeSH Terms] OR "hypertension"[MeSH Terms] OR "blood pressure"[MeSH Terms] OR "hyperuricemia"[MeSH Terms] OR "obesity"[MeSH Terms] OR "uric acid"[MeSH Terms] OR "body mass index"[MeSH Terms] OR "body height"[MeSH Terms] OR "body

weight"[MeSH Terms] OR "waist circumference"[MeSH Terms] OR "waist-hip ratio"[MeSH Terms] OR "waist-height ratio"[MeSH Terms] OR "glomerular filtration rate"[MeSH Terms] OR "albuminuria"[MeSH Terms] OR "proteinuria"[MeSH Terms] OR "diabetic nephropathies"[MeSH Terms] OR "diabetic retinopathy"[MeSH Terms] OR "peripheral arterial disease"[MeSH Terms] OR "cardiovascular diseases"[MeSH Terms] OR "c-reactive protein"[MeSH Terms] OR "sex"[MeSH Terms] OR "gender identity"[MeSH Terms] OR "smoking"[MeSH Terms] OR "drinking behavior"[MeSH Terms] OR "life style"[MeSH Terms] OR "age of onset"[MeSH Terms] OR "risk factor*" OR "hyperglycemia" OR "hyperglycaemia" OR "glycosylated hemoglobin A1c" OR "HbA1c" OR "glycemic control" OR "glycaemic control" OR "hyperlipidemia*" OR "lipid*" OR "triglyceride*" OR "triacylglycerol*" OR "cholesterol" OR "hypertension" OR "blood pressure" OR "hyperuricemia" OR "uric acid" OR "bilirubin" OR "obesity" OR "body mass index" OR "BMI" OR "height" OR "weight" OR "waist circumference" OR "waist hip ratio" OR "waist to hip ratio" OR "waist height ratio" OR "waist to height ratio" OR "glomerular filtration rate" OR "eGFR" OR "creatinine clearance rate" OR "serum creatinine" OR "albuminuria" OR "microalbuminuria" OR "macroalbuminuria" OR "albumin excretion rate" OR "urinary albumin" OR "albumin to creatinine ratio" OR "albumin creatinine ratio" OR "diabetic retinopath*" OR "peripheral arterial disease*" OR "cardiovascular disease*" OR "crp" OR "sex" OR "gender" OR "gender identity" OR "smoking" OR "drinking" OR "physical activity" OR "life style" OR "lifestyle" OR "age" OR "age of onset" OR "race" OR "region" OR "family medical history" OR "family history" OR "disease course" OR "duration"

#4 "cohort studies"[MeSH Terms] OR "prospective studies"[MeSH Terms] OR "follow-up studies"[MeSH Terms] OR "longitudinal studies"[MeSH Terms] OR "case-control studies"[MeSH Terms] OR "cohort stud*" OR "prospective stud*" OR "follow-up stud*" OR "longitudinal stud*" OR "case-control stud*" OR "cross-sectional studies"[MeSH Terms] OR "cross-sectional stud*"

#5 #1 AND #2 AND #3 AND #4

Detailed search strategy in Embase

#1 'non insulin dependent diabetes mellitus'/exp OR 'non insulin dependent diabetes mellitus' OR 'type 2 diabetes mellitus'

#2 'diabetic neuropathy'/exp OR 'diabetic neuropath*' OR 'diabetic peripheral neuropath*' OR 'diabetic sensorimotor polyneuropath*' OR 'distal symmetrical polyneuropath*' OR 'somatosensory disorder'/exp OR ('skin tingling'/exp OR 'tingling') OR ('unsteadiness'/exp OR 'unsteadiness') OR ('achilles reflex'/exp OR 'achilles reflex' OR "ankle reflex'') OR ('hyporeflexia'/exp OR 'hyporeflexia') OR ('temperature sense'/exp OR 'temperature sens*' OR 'temperature perception') OR ('vibration sense'/exp OR 'vibration sens*' OR 'vibration perception') OR ('nerve conduction'/exp OR 'nerve conduction' OR 'neural conduction') OR 'hypesthesia' OR 'paresthesia' OR 'hyperalgesia' OR 'hyperesthesia' OR 'numbness' OR 'prickling' OR 'burning pain' OR 'touch sensation' OR 'touch perception' OR 'Rydel-Seiffer fork' OR 'pinprick sens*' OR 'pinprick perception' OR "10-g monofilament test" OR "Semmes-Weinstein"

#3 'risk factor'/exp OR 'risk factor*' OR ('hyperglycemia'/exp OR 'hyperglycemia' OR 'hyperglycaemia') OR ('hemoglobin A1c'/exp OR 'hemoglobin A1c' OR 'HbA1c') OR ('glycemic control'/exp OR 'glycemic control' OR 'glycaemic control') OR ('hyperlipidemia'/exp OR 'hyperlipidemia*' OR 'lipid*') OR ('triacylglycerol'/exp OR 'triacylglycerol*' OR 'triglyceride*') OR ('cholesterol'/exp OR 'cholesterol') OR ('hypertension'/exp OR 'hypertension') OR ('blood pressure'/exp OR 'blood pressure') OR ('hyperuricemia'/exp OR 'hyperuricemia') OR ('uric acid'/exp OR 'uric acid') OR ('bilirubin'/exp OR 'bilirubin') OR ('obesity'/exp OR 'obesity') OR ('body mass'/exp OR 'body mass index' OR 'BMI')

OR ('height'/exp OR 'height') OR ('body weight'/exp OR 'weight') OR ('waist circumference'/exp OR 'waist circumference') OR ('waist hip ratio'/exp OR 'waist hip ratio' OR 'waist to hip ratio') OR ('waist to height ratio'/exp OR 'waist to height ratio' OR 'waist height ratio') OR ('glomerulus filtration rate'/exp OR 'glomerulus filtration rate' OR 'eGFR' OR 'creatinine clearance rate') OR ('creatinine blood level'/exp OR 'creatinine blood level' OR 'serum creatinine') OR ('albuminuria'/exp OR 'albuminuria') OR ('proteinuria'/exp OR 'proteinuria') OR ('microalbuminuria'/exp OR 'microalbuminuria') OR ('macroalbuminuria'/exp OR 'macroalbuminuria') OR ('urinary albumin excretion rate'/exp OR 'urinary albumin excretion rate') OR ('albumin excretion rate'/exp OR 'albumin excretion rate' OR 'urinary albumin') OR ('albumin to creatinine ratio'/exp OR 'albumin to creatinine ratio') OR ('albumin creatinine ratio'/exp OR 'albumin creatinine ratio') OR ('diabetic nephropathy'/exp OR 'diabetic nephropath*') OR ('diabetic retinopathy'/exp OR 'diabetic retinopath*') OR ('peripheral occlusive artery disease'/exp OR 'peripheral occlusive artery disease*' OR 'peripheral arterial disease*') OR ('cardiovascular disease'/exp OR 'cardiovascular disease*') OR ('C reactive protein'/exp OR 'C reactive protein' OR 'CRP') OR ('gender and sex'/exp OR 'sex' OR 'gender') OR ('gender identity'/exp OR 'gender identity') OR ('smoking'/exp OR 'smoking') OR ('drinking behavior'/exp OR 'drinking') OR ('physical activity'/exp OR 'physical activity') OR ('lifestyle'/exp OR 'lifestyle' OR 'life style') OR ('age'/exp OR 'age') OR ('race'/exp OR 'race' OR 'region') OR ('family history'/exp OR 'family history' OR 'family medical history') OR ('disease course'/exp OR 'disease course' OR 'duration')

#4 'cohort analysis'/exp OR 'cohort stud*' OR ('prospective study'/exp OR 'prospective stud*') OR ('follow up'/exp OR "follow-up stud*") OR ('longitudinal study'/exp OR 'longitudinal stud*') OR ('case control study'/exp OR 'case-control stud*') OR ('cross-sectional study'/exp OR 'cross-sectional stud*')

#5 #1 AND #2 AND #3 AND #4

Detailed search strategy in the Cochrane Library

#1 MeSH descriptor: [Diabetes Mellitus, Type 2] explode all trees

#2 "type 2 diabetes mellitus" (Word variations have been searched)

#3 #1 or #2

#4 MeSH descriptor: [diabetic neuropathies] or [somatosensory disorders] or [reflex, abnormal] or [neural conduction] explode all trees

#5 "diabetic neuropath*" or "diabetic peripheral neuropath*" or "diabetic sensorimotor polyneuropath*" or "distal symmetrical polyneuropath*" or "hypesthesia" or "paresthesia" or "hyperalgesia" or "hyperesthesia" or "numbness" or "tingling" or "unsteadiness" or "prickling" or "burning pain" or "ankle reflex" or "achilles reflex" or "hyporeflexia" or "touch sens*" or "touch perception" or "temperature sens*" or "temperature perception" or "vibration sens*" or "vibration perception" or "Rydel-Seiffer fork" or "pinprick sens*" or "pinprick perception" or "10-g monofilament test" or "Semmes-Weinstein" or "neural conduction" or "nerve conduction" (Word variations have been searched)

#6 #4 or #5

#7 MeSH descriptor: [Risk Factors] or [hyperglycemia] or [glycated hemoglobin A] or [hyperlipidemias] or [lipids] or [triglycerides] or [cholesterol] or [Hypertension] or [Blood Pressure] or [hyperuricemia] or [Uric Acid] or [Obesity] or [body mass index] or [body height] or [body weight]

or [Waist Circumference] or [waist-hip ratio] or [waist-height ratio] or [glomerular filtration rate] or [albuminuria] or [proteinuria] or [diabetic nephropathies] or [diabetic retinopathy] or [peripheral arterial disease] or [cardiovascular diseases] or [c-reactive protein] or [sex] or [gender identity] or [smoking] or [drinking behavior] or [life style] or [age of onset] explode all trees

#8 "risk factor*" or "hyperglycemia" or "hyperglycaemia" or "glycosylated hemoglobin A1c" or "HbA1c" or "glycemic control" or "glycaemic control" or "hyperlipidemia*" or "triglyceride*" or "triacylglycerol*" or "cholesterol" or "hypertension" or "blood pressure" or "hyperuricemia" or "uric acid" or "bilirubin" or "obesity" or "body mass index" OR "BMI" or "height" or "weight" or "waist circumference" or "waist hip ratio" or "waist to hip ratio" or "waist height ratio" or "waist to height ratio" or "glomerular filtration rate" or "eGFR" or "creatinine clearance rate" or "serum creatinine" or "albuminuria" or "proteinuria" or "microalbuminuria" or "macroalbuminuria" or "albumin excretion rate" or "urinary albumin" or "albumin to creatinine ratio" or "albumin creatinine ratio" or "diabetic nephropath*" or "diabetic retinopath*" or "peripheral arterial disease*" or "cardiovascular disease*" or "c-reactive protein" or "CRP" or "sex" or "gender" or "gender identity" or "smoking" or "drinking" or "life style" or "lifestyle" or "physical activity" or "age" or "age of onset" or "race" or "region" or "family medical history" or "family history" or "disease course" or "duration" (Word variations have been searched)

#9 #7 or #8

#10 MeSH descriptor: [Cohort Studies] or [prospective studies] or [follow-up studies] or [longitudinal studies] or [case-control studies] or [cross-sectional studies] explode all trees

#11 "cohort stud*" or "prospective stud*" or "follow-up stud*" or "longitudinal stud*" or "case-control stud*" or "cross-sectional stud*"

#12 #10 or #11

#13 #3 and #6 and #9 and #12

Inclusion and exclusion criteria

We included both published retrospective and prospective cohort studies, in which the risk factors for initiation of diabetic peripheral neuropathy (DPN) were examined and reported as risk ratios (RRs) with 95% confidence limits (CIs). Patients with type 2 diabetes mellitus (T2DM) and without DPN at baseline were eligible. We excluded studies in which the risk factors were novel predictive markers of DPN, such as genomics and proteomics, and it is difficult to be widely used in clinical practice because of their high cost and instability. Studies were also excluded when data were incomplete or severely missing and unavailable. For articles based on the same cohort population, the best one was selected for inclusion according to data integrity and the follow-up interval.

Definition

T2DM was defined as the following conditions: fasting plasma glucose (FPG) level ≥7.0 mmol/l (126 mg/dl), 2-h plasma glucose level ≥11.1 mmol/l (199 mg/dl) during an oral glucose tolerance test (OGTT), hemoglobin A1c (HbA1c) level ≥6.5% (48mmol/mol), treatment with antidiabetic drugs, self-reported diabetes, or using administrative data coding algorithms[3,4]. The diagnosis of DPN was in line with the guideline provided by 2010 Toronto Consensus(3), as follows: (1) T2DM is clearly diagnosed; (2) The neuropathy is consistent with DPN at or after the diagnosis of T2DM; (3) Any two

or more of the following occur: distal paresthesia (positive or negative), decreased temperature sensation, abnormal 10-g nylon monofilament test, abnormal vibration sensation (128-Hz tuning fork), decreased or absent ankle reflexes. (4) Any two or more abnormalities (prolonged conduction latency, decreased amplitude, and/or slowed speed) in neurophysiological tests exist; (5) The neuropathy caused by other reasons should be excluded.

Outcome

Initiation of DPN.

Ethics Statement

The study presented here was approved by the Ethics Committee of Tianjin Medical University Metabolic Diseases Hospital and Tianjin Institute of Endocrinology. It was agreed to waive the requirement for informed consent, as this study was a retrospective study, and data came from articles published in databases.

Data extraction and quality assessment

According to the search strategy, we strictly searched published studies on the risk factors for DPN and screened the researches according to the inclusion and exclusion criteria. First, by reading the titles and abstracts, and manually searching the relevant references, a preliminary screening of the articles was carried out; then, the full texts of the studies included in the preliminary screening were read through, and the final screening of the literature was conducted to determine the final included studies. In the final screening process, a literature information table was developed. The following information were extracted: title, first author, publication date, country or region, details of the study design, sample size, population source, and baseline data, including gender, age, duration of diabetes mellitus (DM), follow-up, end-point events, number of new cases, risk factors for initiation of DPN and the RR/HR (represented as RR in this meta-analysis) with 95% CI. Baseline characteristics and risk factors of the 18 cohort studies are shown in Supplementary Table 1. The quality of all included cohorts was assessed using the Newcastle-Ottawa Scale (NOS)(4). NOS has 3 columns: selection, comparability and outcome, with a total of 9 points. NOS score range from 0 to 9. Articles with a score of 7 or higher was considered high quality. Data extraction and quality assessment are shown in Supplementary Table 2.

Statistical analysis

The risk ratio (RR) value and 95% confidence interval (CI) of each risk factor were extracted from the included cohorts, and then pooled to screen out risk factors according to the heterogeneity across studies. Heterogeneity test was analyzed by Q test, and measured by I2 value. When there was statistically significant heterogeneity (P value < 0.10 or I2 value > 50%), the pooled RR and 95% CI were generated by a random effects model, otherwise by a fixed effects model. Subgroup analyses were performed according to the magnitude of the increase in continuous variables. Continuous variables included age (years, increment by 1 vs 5-10), body mass index (BMI) (kg/m2, increment by 1-5), and duration of DM (years, increment by 1 vs 5-10). Sensitivity analyses were conducted to evaluate the robustness of the results after a single study was omitted. Publication biases were determined using Begg's and Egger's linear regression tests, and the latter one prevailed if the two results were inconsistent. All tests were considered statistical significance at two-tailed P value < 0.05, except for heterogeneity test and publication bias were at P value < 0.1. Statistical analyses were performed with Stata software (version 12.0 StataCorp, College Station, TX).

Results

Search results

1626 articles from Pubmed, 1710 from Embase and 86 from the Corchrane Library were found. 385 duplicated articles were excluded. 3037 articles were excluded after carefully reviewing the titles and abstracts. Of the 417 articles that underwent full-text evaluation and 15 additional articles from hand-searching, 18 cohort studies met our inclusion criteria.

Characteristics and quality of included studies

Of the 18 included studies, 13 were prospective cohorts and 5 were retrospective cohorts. There were 95,604 patients with T2DM, who were mainly from 19 countries and regions. Of those, 50% were from Asia, 22.22% were from Europe, 22.22% were from America, and 5.56% were from Oceania. A total of 95,604 patients with T2DM were included in the derivation cohort, with age between 35 and 79 years old, male accounting for 49.6%, and duration of DM ranging 0-19 years. The follow-up was 0 to 19 years, and 19,399 DPN events were observed, with an estimated incidence of 20.3%. The characteristics of all these 18 cohorts were shown in Supplementary Table 1. All the 18 cohort studies were of high quality as assessed by the Newcastle-Ottawa Scale (NOS) (provided in Supplementary Table 2).

Risk factors of DPN in patients with T2DM

There were 24 risk factors available from these studies, including age, gender, marital status, smoking, height, BMI, waist circumference (WC), duration of DM, FPG, HbA1c, total cholesterol (TC), triglyceride (TG), high density lipoprotein (HDL-c), low density lipoprotein (LDL-c), systolic blood pressure (SBP), diastolic blood pressure (DBP), c-reactive protein (CRP) , estimated glomerular filtration rate (eGFR), hypertension, diabetic retinopathy (DR), diabetic kidney disease (DKD), cardiovascular disease (CVD), insulin, and statins. (Shown in Supplementary Table 3).

11 risk factors were involved in DPN onset. The risk stratification methods were carefully selected by subgroup or sensitivity analyses, which were most reasonable considering the feasibility and convenience of clinical practice. These 11 risk factors included in the final model were as follows: age incremented by 1 year (RR 1.02, 95%CI 1.01-1.03, P = 0.001), smoking (RR 1.43, 95%CI 1.29-1.59, P < 0.001), BMI incremented by 1-5 kg/m2 (RR 1.18, 95%CI 1.02-1.37, P = 0.030), duration of diabetes incremented by 5-10 years (RR 1.39, 95%CI 1.21-1.60, P < 0.001), HbA1c incremented by1% (RR 1.14, 95%CI 1.08-1.19, P < 0.001), low HDL-c (RR 1.34, 95%CI 1.13-1.59, P = 0.001), high triglyceride (HTG) (RR 1.34, 95%CI 1.19-1.51, P < 0.001), hypertension (HTN) (RR 1.35, 95%CI 1.08-1.68, P = 0.008), DR (RR 2.05, 95%CI 1.25-3.37, P = 0.005), DKD (RR 1.91, 95%CI 1.32-2.77, P = 0.001), and CVD (RR 1.66, 95%CI 1.33-2.08, P < 0.001). Details of 11 risk factors in the final model are shown in Supplementary Figure 1-11. One risk factor exhibited bias—duration of diabetes. The results of Begg's (Z, Pr > |z|) and Egger's (t, P) linear regression tests were (2.35, 0.019) and (5.99, 0.001), respectively. The overall publication bias is relatively good. All publication bias of DPN risk factors is shown in Supplementary Table 4.

2 Supplementary Figures and Tables

Supplementary Table 1. Baseline characteristics and risk factors of the 18 cohorts

First author/Year/Country or region (continent)	Cohort design/period	Source of cohort	Sample size (male%)	Age (years)	Duration of DM (years)	Follow-up (years)	Risk Factors of DPN
Pek(5)/2020/Singapore (Southeast Asia)	Prospective/ 2011-2014	SMART2D study	1250 (48.9)	56.3±10.4	10.1±8.2	3	Age, gender, race, duration of DM, HbA1c, SBP, eGFR, UACR, DR, PAD
Teliti(6)/2018/Italy(Europe)	Retrospective/1999-2016	ICS	900 (57.2)	67.0±10.0	10.35±8.37	2	Gender, duration of DM, HbA1c, DR, macroangiopathy$^{\Delta}$
Hafshejani(7)/2018/Iran (Asia)	Prospective/ 2007-2016	Diabetic Patient Care Program	1874 (44.8)	56.5±15.7	NA	2	Age, gender, residence, marital status, education, smoking, BMI, HbA1c, SBP, DBP, TC, HDL-c, LDL-c
Fawwad(8)/2018/Pakistan (South Asia)	Retrospective/2005-2016	Pakistan T2D cohort study	4633 (50.4)	50.7±10.8	NA	12	Duration of DM, HbA1c, hypertension
Aryan(9)/2018/Iran (Asia)	Prospective/ 2005-2016	MetsCoM cohort study	1301 (47.6)	55.1 ± 0.3	NA	7.5	CRP
Andersen(10)/2018/Germany (Europe)	Prospective/ 2001-2006	ADDITION	1256 (58.5)	60.8 (55.6-65.6)	NA	13	Height, weight, BMI, WC, HbA1c, TG, TC, HDL-c, LDL-c, SBP, DBP
Yang(11)/2017/China (Asia)	Retrospective/2002-2011	National Diabetes Care Management Program	36152 (49.0)	NA	NA	7.23	HbA1c, FPG-CV
Yang(12)/2015/China (Asia)	Retrospective/2002-2011	Taiwan Diabetes Study	37375 (49.0)	≥30	NA	7	HbA1c, TG, HDL-c, LDL-c, eGFR, hypertension, overweight
Cardoso(13)/2015/Brazil (Latin America)	Prospective/ 2004-2014	Rio de Janeiro T2D Cohort Study	477 (36.3)	59.3±9.0	8 (3–15)	6.2	Height, HbA1c, hypertension, CKD, aortosclerosis
Muñoz(14)/2014/Spain (Europe)	Prospective/ 2002–2012	North Catalonia Diabetes Study	267 (62.9)	57.2±8.0	5 (3–9)	10	Age, gender, duration of DM, LDL-c, CVD$^{\Delta\Delta}$
Cardoso(15)/2008/Brazil	Prospective/ 1994-2001	Brazilian T2D patients	471 (65.8)	60.5±11.1	9.3±7.3	2	Duration of DM, FPG, HDL-c, left

(Latin America)							ventricular mass
Abougalambou (16)/2012/Malaysia (Southeast Asia)	Prospective/ 2008-2009	HUSM	1077 (45.2)	≥18	NA	NA	Duration of DM, HbA1c, eGFR, DR
Davis(17)/2008/Australia (Oceania)	Prospective/ 1993-2001	FDS	395 (51.4)	61.5±11.2	3.0 (0.7-7.0)	5	Age, marital status, race, DR, statins
Sands(18)/1997/USA (North America)	Prospective/ 1984-1992	San Luis Valley Diabetes Study	231 (NA)	range 20-74	NA	4.7（2.0-6.6）	Age, gender, race, duration of DM, HbA1c, CVD[ΔΔ], smoking, insulin
Chuengsamarn (19)/2017Thailand (Southeast Asia)	Prospective/ 2014-2015	Diabetic Center of HRH Princess Maha	608 (29.6)	57.79±13.39	7.85±8.12	1	CRP
Cho(20)/2014/ Korea (Asia)	Retrospective /2006–2012	Korean T2D cohort study	48 (81.3)	64.88±7.14	14.93±6.91	6	BMI, HDL-c, LDL-c
Christensen(21)/2020/Denmark (Europe)	Prospective/ 2010-2016	DD2	5249 (58)	65 (57-72)	4.6 (3.5-5.7)	2.8	BMI, WC, WHt, HbA1c, TG, TC, HDL-c, LDL-c, CRP, C-P, SBP, DBP, exercise, smoking
Sayah(22)/2015/Canada (North America)	Prospective/ 2011-2013	ABCD	2040 (55)	64.4±10.7	12	2	Residence, duration of DM, HbA1c, hyperlipemia, CVD[ΔΔ], cerebrovascular disease (stroke/TIA), depression

Note: [Δ] Macroangiopathy included previous myocardial infarction, stroke, diabetic foot ulcers or gangrene, amputation, surgery in coronary, carotid, and peripheral artery. [ΔΔ] CVD, cardiovascular disease. CVD included angina, previous myocardial infarction, or electrocardiographic manifestations of coronary ischemia. NA, not available.

Supplementary Table 2. Newcastle-Ottawa Quality Assessment Scale of the 18 cohort

First author/Year	Selection				Comparability	Outcome			Total scores
	Representativeness of the exposed cohort	Selection of the non-exposed cohort	Ascertainment of exposure	Demonstration that outcome of interest was not present at start of study	Comparability of cohorts on the basis of the design or analysis	Assessment of outcome	Was follow-up long enough for outcomes to occur	Adequacy of follow up of cohorts	
Pek/2020	☆	☆	☆	☆	☆☆	☆	☆	☆	9
Teliti/2018	☆	☆	☆	☆	☆☆	☆	☆	☆	9
Hafshejani/2018	☆	☆	☆	☆	☆☆	☆	☆	☆	9
Fawwad/2018	☆	☆	☆	☆	☆☆	☆	☆	☆	9
Aryan/2018	☆	☆	☆	☆	☆☆	☆	☆	☆	9
Andersen/2018	☆	☆	☆	☆	☆☆	☆	☆	☆	9
Yang/2017	☆	☆	☆	☆	☆☆	☆	☆	☆	9
Yang/2015	☆	☆	☆	☆	☆☆	☆	☆	☆	9
Cardoso/2015	☆	☆	☆	☆	☆☆	☆	☆	☆	9
Muñoz/2014	☆	☆	☆	☆	☆☆	☆	☆	☆	9
Cardoso/2008	☆	☆	☆	☆	☆	☆	☆	☆	8
Abougalambou/2012	☆	☆	☆	☆	☆☆	☆	☆	☆	9
Davis/2008	☆	☆	☆	☆	☆☆	☆	☆	☆	9
Sands/1997	☆	☆	☆	☆	☆☆	☆	☆	☆	9
Chuengsamarn/2017	☆	☆	☆	☆	☆☆	☆	☆	☆	9
Cho/2014	☆	☆	☆	☆	☆☆	☆	☆	☆	9
Christensen/2020	☆	☆	☆	-	☆☆	☆	☆	☆	8
Sayah/2015	☆	☆	☆	☆	☆☆	☆	☆	☆	9

Supplementary Table 3. 11 risk factors included in the systematic review and meta-analysis.

Risk factors	First author/Year	Sample size	No. of ESRD	Definition of risk factor	RR/HR	95%CI	*P*
Age	Pek/2020	1250	134	Increment by 1 year	1.013	0.992–1.035	0.214
Age	Hafshejani /2018	1874	337	Increment by 1 year	1.020	1.009-1.06	0.001
Age	Munoz/2016	267	49	Increment by 1 year	1.03	0.98–1.07	0.22
Age	Abougalambou/2012	1077	589	Increment by 1 year	1.02	1.01- 1.04	<0.001
Age	Sands/1997	231	66	Increment by 10 years	1.0	0.7-1.4	0.92
Age	Davis/2008	395	248	Increment by 10 years	1.86	1.60–2.15	<0.001
Gender	Pek/2020	1250	134	male	1.283	0.886–1.860	0.187
Gender	Teliti/2018	900	123	male	1.78	1.133–2.799	<0.05
Gender	Hafshejani/2018	1874	337	male	3.66	1.15-11.67	0.028
Gender	Muñoz/2016	267	49	female	1.43	0.71–2.88	0.31
Gender	Sands/1997	231	66	female	1.7	0.9-3.3	0.13
Marital status	Hafshejani/2018	1874	337	Married vs single	2.18	1.24-3.83	0.007
Marital status	Hafshejani/2018	1874	337	Divorced or widowed (vs single)	3.02	1.61-5.65	0.001
Marital status	Davis/2008	395	248	Married or cohabiting relationship	0.75	0.57–0.98	0.038
Smoking	Sands/1997	231	66	Current smoking	2.2	0.99-4.7	0.05
Smoking	Hafshejani/2018	1874	337	Smoking	1.21	0.87-1.68	0.246
Smoking	Christensen/2020	3437	608	Current smoking	1.5	1.24-1.81	NA
Smoking	Sayah/2015	2040	372	Current smoking	1.4	0.9-2.1	0.1
Smoking	Christense/2020	3437	608	Quitting smoking	1.39	1.18-1.64	NA
Smoking	Sayah/2015	2040	372	Quitting smoking	1.5	1.2-2.0	0.001
Smoking	Christensen/2020	5249	938	Current smoking	1.39	1.18-1.64	NA
Smoking	Christensen/2020	5249	938	Quitting smoking	1.5	1.24-1.81	NA
Height	Cardoso/2008	471	73	Increment by 10 cm	1.25	0.79-1.98	NS
Height	Andersen/2018	1256	78	Increment by 5 cm	0.97	0.83-1.12	NS
WC	Andersen/2018	1256	78	Increment by 5 cm	1.14	1.05-1.24	<0.05
WC	Christensen/2020	5239	937	94-101/80-87 (vs <94/80 cm)	1.67	1.16-2.41	NA
WC	Christensen/2020	5239	937	≥102/88 (vs <94/80 cm)	2.45	1.76-3.40	NA
BMI	Hafshejani/2018	1874	337	Increment by 1 kg/m^2	1.03	1-1.06	0.032
BMI	Yang/2015	37375	8386	Overweight (HbA1c≥7%)	1.34	1.24-1.46	<0.001
BMI	Yang/2015	37375	8386	Overweight (HbA1c<7%)	1.12	1.02-1.23	<0.05

BMI	Christensen/2020	4542	919	25-29 (vs <25kg/m^2)	1.3	0.97-1.75	NA
BMI	Christensen/2020	4542	919	30-34 (vs <25 kg/m^2)	1.65	1.23-2.20	NA
BMI	Christensen/2020	4542	919	≥35 (vs <25 kg/m^2)	1.88	1.40-2.52	NA
BMI	Cho/2014	39	6	BMI>25 kg/m^2	2.59	0.46-14.34	0.285
BMI	Andersen/2018	1256	78	Increment by 2 kg/m^2	1.14	1.06-1.23	<0.05
BMI	Christensen/2020	4542	919	25-29 (vs <25 kg/m^2)	1.3	0.97-1.75	NA
BMI	Christensen/2020	4542	919	30-34 (vs <25 kg/m^2)	1.65	1.23-2.20	NA
BMI	Christensen/2020	4542	919	≥35 (vs <25 kg/m^2)	1.88	1.40-2.52	NA
Duration of DM	Pek/2020	1250	134	Increment by 1 year	1.025	1.004-1.047	0.017
Duration of DM	Teliti/2018	900	123	Increment by 1 year	1.046	1.022–1.071	<0.0001
Duration of DM	Fawwad/2018	4633	492	5-10 (vs <5 years)	1.33	1.13-1.58	0.0006
Duration of DM	Fawwad/2018	4633	492	>10 (vs <5 years)	1.76	1.47-2.10	<0.0001
Duration of DM	Muñoz/2016	267	49	Increment by 1 year	1.05	1.00–1.10	0.04
Duration of DM	Abougalambou/2012	1077	589	Increment by 1 year	1.03	1.01- 1.05	0.003
Duration of DM	Cardoso/2008	471	73	Increment by 1SD (7.3year)	1.38	1.10–1.72	0.005
Duration of DM	Sands/1997	231	66	Increment by 5 years	1.3	0.98-1.6	0.07
Duration of DM	Sayah/2015	2040	372	≥10 years	1.8	1.4-2.3	<0.001
FPG	Hafshejani/2018	1874	337	Increment by 1 mmol/L	1.02	1.01-1.03	0.001
FPG	Cardoso/2008	471	73	Increment by 1-SD (0.9 mmol/L)	1.33	1.08–1.63	0.007
HbA1c	Pek/2020	1250	134	Increment by 1%	1.148	0.988–1.333	0.071
HbA1c	Teliti/2018	900	123	Increment by 1%	1.278	1.022–1.598	<0.05
HbA1c	Hafshejani/2018	1874	337	Increment by 1%	1.10	1.04-1.16	0.001
HbA1c	Fawwad/2018	4633	492	Increment by 1%	1.11	0.894-1.405	0.328
HbA1c	Andersen/2018	1256	78	Increment by 1%	0.93	0.75-1.15	NS
HbA1c	Yang/2017	36152	7219	Increment by 1%	1.13	1.07–1.20	<0.001
HbA1c	Yang/2015	37375	8386	8-9 (vs 6-7%)	1.30	1.21-1.40	<0.001
HbA1c	Yang/2015	37375	8386	9-10 (vs 6-7%)	1.32	1.22-1.43	<0.001
HbA1c	Yang/2015	37375	8386	≥10 (vs 6-7%)	1.62	1.51-1.74	<0.001
HbA1c	Yang/2015	37375	8386	<6 (vs 6-7%)	1.05	0.96-1.16	NS
HbA1c	Cardoso/2015	342	50	Increment by 1%	1.04	0.85-1.28	NS
HbA1c	Abougalambou/2012	1077	589	Increment by 1%	1.13	1.07-1.20	<0.001
HbA1c	Sands/1997	231	66	Increment by 1%	1.1	0.8-1.5	0.75
TC	Hafshejani/2018	1874	337	Increment by 1mmol/L	1.002	0.999-1.004	0.066

TC	Andersen/2018	1256	78	Increment by 0. 5 mmol/L	0.9	0.80-1.01	NS
TC	Christensen/2020	2267	417	≥4.3 mmol/L	1.04	0.87-1.24	NA
HDL-c	Hafshejani/2018	1874	337	Increment by 1 mmol/L	0.999	0.989-1.008	0.871
HDL-c	Andersen/2018	1256	78	Increment by 0.25 mmol/L	0.82	0.69-0.99	<0.05
HDL-c	Yang/2015	37375	8386	<50 (female)/40(male) mg/dL (HbA1c≥7%)	1.42	1.32-1.53	<0.001
HDL-c	Yang/2015	37375	8386	<50 (female)/40(male) mg/dL (HbA1c<7%)	1.18	1.08-1.29	<0.001
HDL-c	Cardoso/2008	471	73	Increment by 1SD (0.27 mmol/L)	0.66	0.51–0.85	0.001
HDL-c	Christensen/2020	2274	417	<1.0 (male)/1.2 (female) mmol/L	1.35	1.12-1.62	NA
HDL-c	Cho/2014	39	6	<45 mg/dL	5.292	1.001-27.989	0.050
HDL-c	Cho/2014	39	6	<45 mg/dL	5.292	1.001-27.989	0.050
LDL-c	Hafshejani/2018	1874	337	Increment by 1 mmol/L	1.002	0.999-1.005	0.071
LDL-c	Andersen/2018	1256	78	Increment by 0.25 mmol/L	0.92	0.86-0.98	<0.05
LDL-c	Yang/2015	37375	8386	≥100 mg/dL	1.38	1.27-1.51	<0.001
LDL-c	Yang/2015	37375	8386	≥100 mg/dL	1.11	1.01-1.22	<0.05
LDL-c	Muñoz/2016	900	123	Increment by 1 mmol/L	0.98	0.97–0.99	0.01
LDL-c	Cho/2014	39	6	>177 mg/dL	6.129	1.057-35.528	0.043
LDL-c	Christensen/2020	3433	594	1.8-2.5 (vs <1.8mmol/L)	0.97	0.80-1.16	NA
LDL-c	Christensen/2020	3433	594	≥2.6 (vs <1.8mmol/L)	1.09	0.91-1.32	NA
TG	Andersen/2018	1256	78	Increment by 0.5 mmol/L	1.04	0.98-1.09	NS
TG	Yang/2015	37375	8382	≥150 mg/dL	1.45	1.35-1.55	<0.001
TG	Yang/2015	37375	8388	≥150 mg/dL	1.2	1.10-1.31	<0.001
TG	Christensen/2020	3302	585	≥1.7 mmol/L	1.36	1.17-1.59	NA
SBP	Pek/2020	1250	134	Increment by 10 mmHg	1.107	1.001-1.223	0.047
SBP	Hafshejani/2018	1874	337	Increment by 10 mmHg	1.014	1.006-1.023	0.001
SBP	Andersen/2018	1256	78	Increment by 10 mmHg	1.02	0.90-1.16	NS
DBP	Hafshejani/2018	1874	337	Increment by 1 mmHg	1.016	1.007-1.026	0.001

DBP	Andersen/2018	1256	78	Increment by 5 mmHg	0.96	0.83-1.12	NS
Hypertension	Fawwad/2018	4633	492	>130/85 mmHg	1.30	1.136-1.507	0.0002
Hypertension	Yang/2015	37375	8380	≥130/85 mmHg	1.41	1.29-1.54	<0.001
Hypertension	Yang/2015	37375	8387	≥130/85 mmHg	1.16	1.05-1.28	<0.01
Hypertension	Cardoso/2015	342	50	≥130/85 mmHg, with antihypertensive drugs	6.67	0.90-49.9	<0.10
Hypertension	Christensen/2020	3387	599	SBP ≥130 mmHg	0.95	0.82-1.10	NA
Hypertension	Christensen/2020	3387	599	DBP ≥80 mmHg	1.11	0.95-1.29	NA
Hypertension	Sayah/2015	2040	372	≥130/85 mmHg	1.9	1.5-2.5	<0.001
CRP	Chuengsamarn/2017	608	102	0.12-0.99 (vs 3.00-9.45 mg/L)	0.05	0.01–0.22	<0.05
CRP	Chuengsamarn/2017	608	102	1.00-2.99mg/L (vs 3.00-9.45 mg/L)	0.17	0.07–0.43	<0.05
CRP	Aryan/2018	1301	241	Increment by 1-SD (0.058 mg/L)	1.025	1.021-1.029	<0.001
CRP	Christensen/2020	4722	833	1.0-2.9 (vs <1.0 mg/L)	1.12	0.95-1.31	NA
CRP	Christensen/2020	4722	833	≥3.0 (vs <1.0mg/L)	1.66	1.42-1.94	NA
eGFR	Yang/2015	37375	8387	<60 mL/min/1.73 m^2	1.01	0.91-1.11	NS
eGFR	Abougalambou/2012	1077	589	eGFR	1.27	0.97- 1.58	<0.001
eGFR	Pek/2020	1250	134	eGFR	0.996	0.989–1.003	0.217
DR	Pek/2020	1250	134	DR	1.152	0.776–1.709	0.483
DR	Teliti/2018	900	123	DR	3.162	1.874–5.334	<0.0001
DR	Abougalambou/2012	1077	589	DR	3.06	2.34- 4.01	<0.001
DR	Davis/2008	395	248	DR	1.61	1.10–2.37	0.015
DKD	Cardoso/2015	342	50	DKD	2.27	1.16-4.35	<0.05
DKD	Abougalambou/2012	1077	589	DKD	1.77	1.13-2.77	0.011
CVD	Sands/1997	231	66	CVD	0.8	0.3-2.4	0.75
CVD	Muñoz/2016	900	123	CVD	2.32	1.03–5.22	0.04
CVD	Teliti/2018	267	49	CVD	1.637	1.064–2.518	<0.05
CVD	Sayah/2015	2040	372	CVD	1.7	(1.3, 2.3)	<0.001
Insulin	Hafshejani/2018	1874	337	Insulin	1.29	1.02-1.66	0.036
Insulin	Sands/1997	231	66	Insulin	2.0	0.9-4.4	0.09
Statins	Davis/2008	395	248	Statins	0.70	0.49–0.997	0.048
Statins	Hafshejani/2018	1874	337	Statins	1.28	1.03-1.64	0.034

Note: WC, waist circumference; BMI, body mass index; DM, diabetes mellitus; HbA1c, Hemoglobin A1c; SBP, systolic blood pressure; DBP, diastolic blood pressure; FPG, fasting plasma glucose; TC,

total cholesterol; HDL-c, high density lipoprotein cholesterol; LDL-c, low density lipoprotein cholesterol; TG, triglyceride; CRP, C-reactive protein; eGFR, estimated glomerular filtration rate; DR, diabetic retinopathy; DKD, diabetic kidney disease; CVD, cardiovascular disease. NA, not available; NS, not significant.

Supplementary Table 4. Publication bias of risk factors for DPN

Risk factors	No. of studies	Begg's test		Egger's test	
		Z	*P*r>|z|	t	*P*
Age	6	1.50	0.133	1.46	0.219
Smoking	4	0.34	0.734	0.37	0.749
BMI	5	0.24	0.806	3.00	0.058
Duration of diabetes	8	2.35	0.019	5.99	0.001**
HbA1c	10	0.09	0.929	0.31	0.768
HDL-c	3	1.04	0.296	5.83	0.108
TG	2	0.00	1.000	-	-
Hypertension	5	0.73	0.462	1.39	0.258
DR	4	0.34	0.734	-0.55	0.635
DKD	2	0.00	1.000	-	-
CVD	4	0.34	0.734	-0.53	0.648

** P<0.01

Supplementary Table 5. Baseline characteristics of patients in the validation cohort

Variables	Total	Development of DPN		*P*
		No	Yes	
N	462	213	249	
Follow-up (year)	4.29±1.98	3.91±1.96	4.62±1.95	<0.001
Male [n (%)]	315 (68.2%)	153 (71.8%)	162 (65.1%)	0.119
Smoker [n (%)]	200 (43.3%)	81 (38.0%)	119 (47.8%)	0.035
Age (year)	52.4±12.2	47.0±12.5	55.1±10.6	<0.001
Duration of DM (year)	6.0 (3.0, 11.0)	4.0 (1.1, 8.0)	10.0 (5.0, 13.5)	<0.001
BMI (kg/m^2)	27.49±4.43	27.15±4.21	27.77±4.60	0.133
SBP (mmHg)	136±66	130±17	141±88	0.074
DBP (mmHg)	81±11	82±11	81±12	0.437
HbA1c (%)	8.52±1.88	8.09±1.82	8.88±1.85	<0.001
HDL-c (mmol/L)	1.19±0.28	1.22±0.27	1.15±0.29	0.010
TG (mmol/L)	1.63 (1.15, 2.56)	1.55 (1.12, 2.21)	1.81 (1.18, 2.94)	0.009
HTN [n(%)]	236 (51.1%)	96 (45.1%)	140 (56.2%)	0.017
DR [n(%)]	109 (23.6%)	38 (28.5%)	71 (23.6%)	0.007
DKD [n(%)]	120 (26.0%)	37 (17.4%)	83 (33.3%)	<0.001
CVD [n(%)]	224 (48.5%)	59 (27.7%)	165 (66.3%)	<0.001
OAD [n(%)]	427 (92.4%)	198 (93.0%)	229 (92.0%)	0.689
Insulin [n(%)]	269 (58.2%)	117 (54.9%)	152 (61.0%)	0.184
ACEI/ARB [n(%)]	186 (40.3%)	79 (37.1%)	107 (43.0%)	0.199
Statins [n(%)]	183 (39.6%)	76 (35.7%)	107 (43.0%)	0.110

Note: DM, diabetes mellitus; BMI, body mass index; SBP, systolic blood pressure; DBP, diastolic blood pressure; HbA1c, Hemoglobin A1c; HDL-c, high density lipoprotein cholesterol; TG, triglyceride; HTN, hypertension; DR, diabetic retinopathy; DKD, diabetic kidney disease; CVD, cardiovascular disease; OAD, Oral antidiabetic drug; ACEI, angiotensin converting enzyme inhibitor; ARB, angiotensin receptor blocker.

Supplementary Table 6. Risk factors, risk stratification, RRs, 95%CIs, β-coefficients, and risk scores in the DPN risk prediction model.

Risk factors of DPN	Risk stratification	RR (95%CI)	β	Score
Age	increment by 1year	1.02 (1.01-1.03)	0.020	0.2
Smoking #	no/yes	1.43 (1.29-1.59)	0.358	3.5
BMI	increment by 1-5 kg/m^2	1.18 (1.02-1.37)	0.166	1.5
Duration of DM	increment by 5-10 years	1.39 (1.21-1.60)	0.329	3.0
HbA1c	increment by 1%	1.14 (1.08-1.19)	0.131	1.5
Low HDL-c	no/yes	1.34 (1.13-1.59)	0.293	3.0
HTG	no/yes	1.34 (1.19-1.51)	0.293	3.0
HTN	no/yes	1.35 (1.08-1.68)	0.300	3.0
DR	no/yes	2.05 (1.25-3.37)	0.718	7.0
DKD	no/yes	1.91 (1.32-2.77)	0.647	6.5
CVD	no/yes	1.66 (1.33-2.08)	0.507	5.0

Supplementary Table 7. The sensitivity, specificity and Youden index of different cutoff risk scores

Cut-off value	Sensitivity	Specificity	Youden index
12.0	0.960	0.319	0.279
12.5	0.956	0.347	0.303
13.0	0.956	0.404	0.360
13.5	0.940	0.423	0.362
14.0	0.936	0.460	0.396
14.5	0.928	0.507	0.435
15.0	0.912	0.549	0.461
15.5	0.900	0.568	0.468
16.0	0.892	0.610	0.502
16.5	0.884	0.639	0.522
17.0 $^{\Delta}$	0.859	0.671	0.531
17.5	0.831	0.700	0.531
18.0	0.803	0.723	0.526
18.5	0.779	0.728	0.507
19.0	0.763	0.747	0.510
19.5	0.739	0.747	0.486
20.0	0.715	0.765	0.480
20.5	0.695	0.789	0.484
21.0	0.667	0.822	0.488
21.5	0.659	0.855	0.513
22.0	0.639	0.864	0.502
22.5	0.623	0.869	0.491
23.0	0.566	0.878	0.444
23.5	0.534	0.883	0.417
24.0	0.518	0.887	0.405
24.5	0.494	0.897	0.391
25.0	0.462	0.906	0.368

Note: $^{\Delta}$ Optimal cutoff point.

Supplementary Figure 1. Age

A

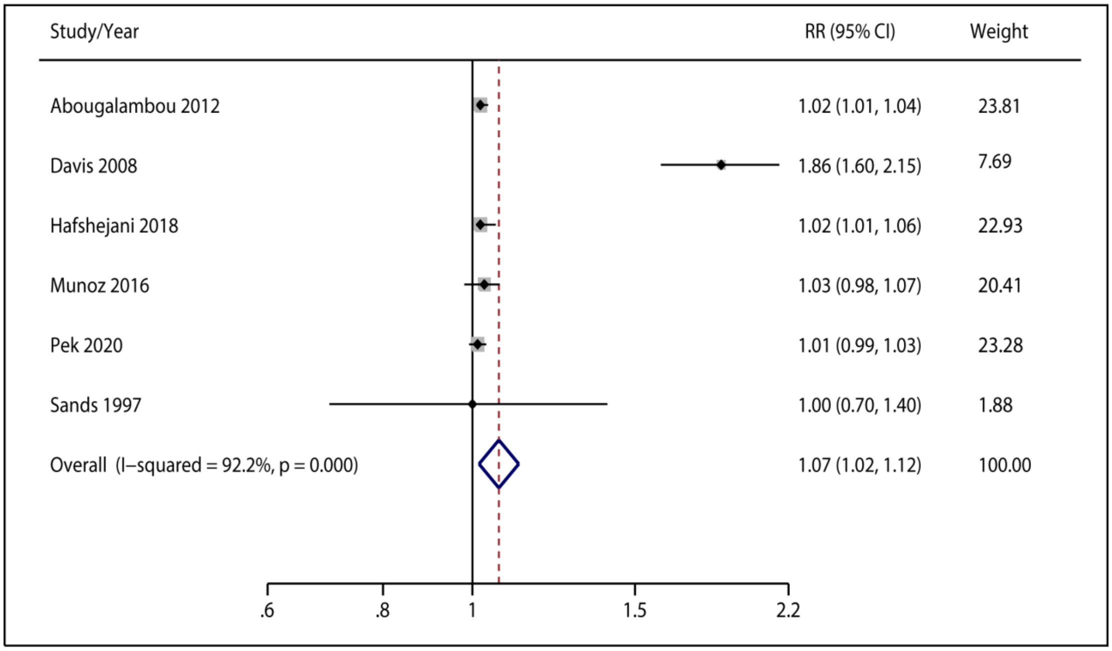

B

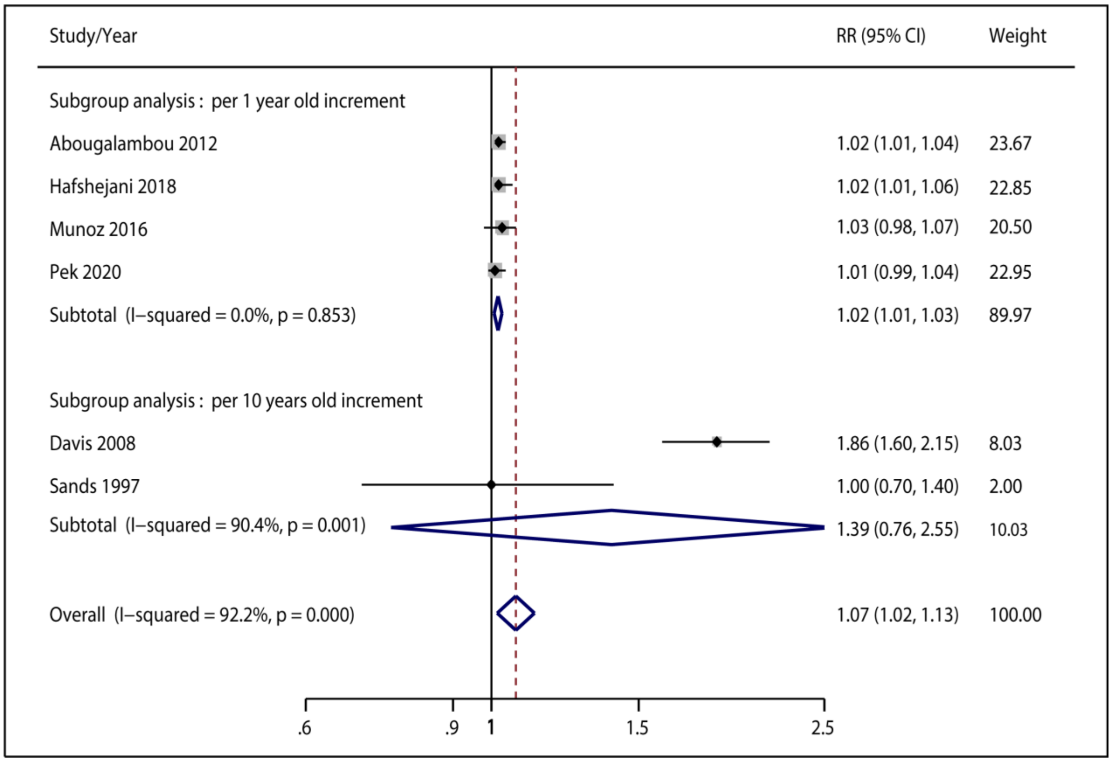

Figure 1- A. Association of age with diabetic peripheral neuropathy; B. Subgroup analysis of association of age with diabetic peripheral neuropathy.

Supplementary Figure 2. Smoking

A

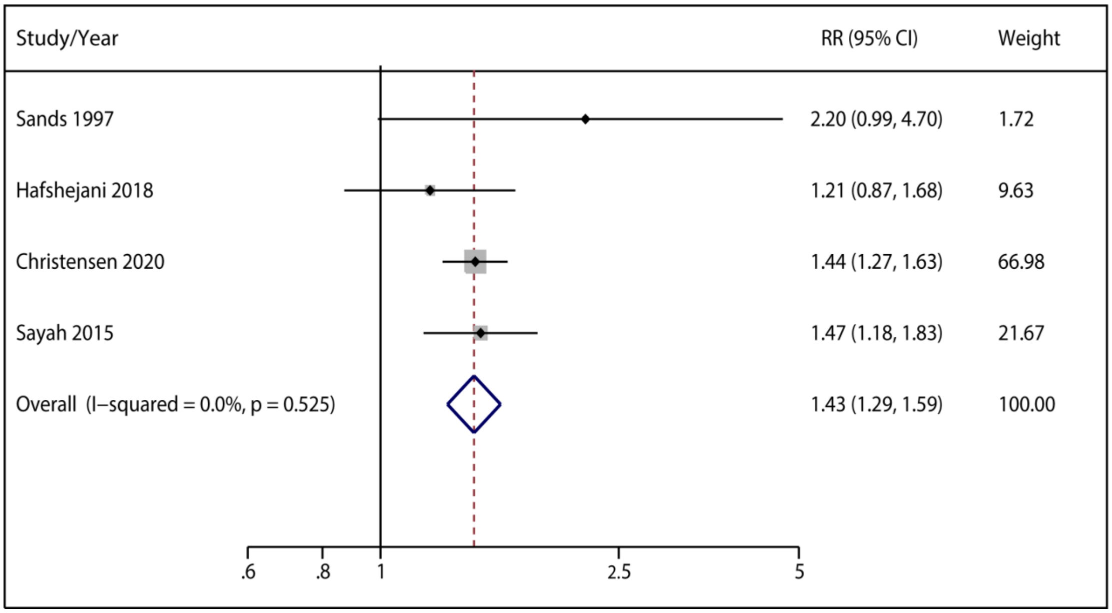

B

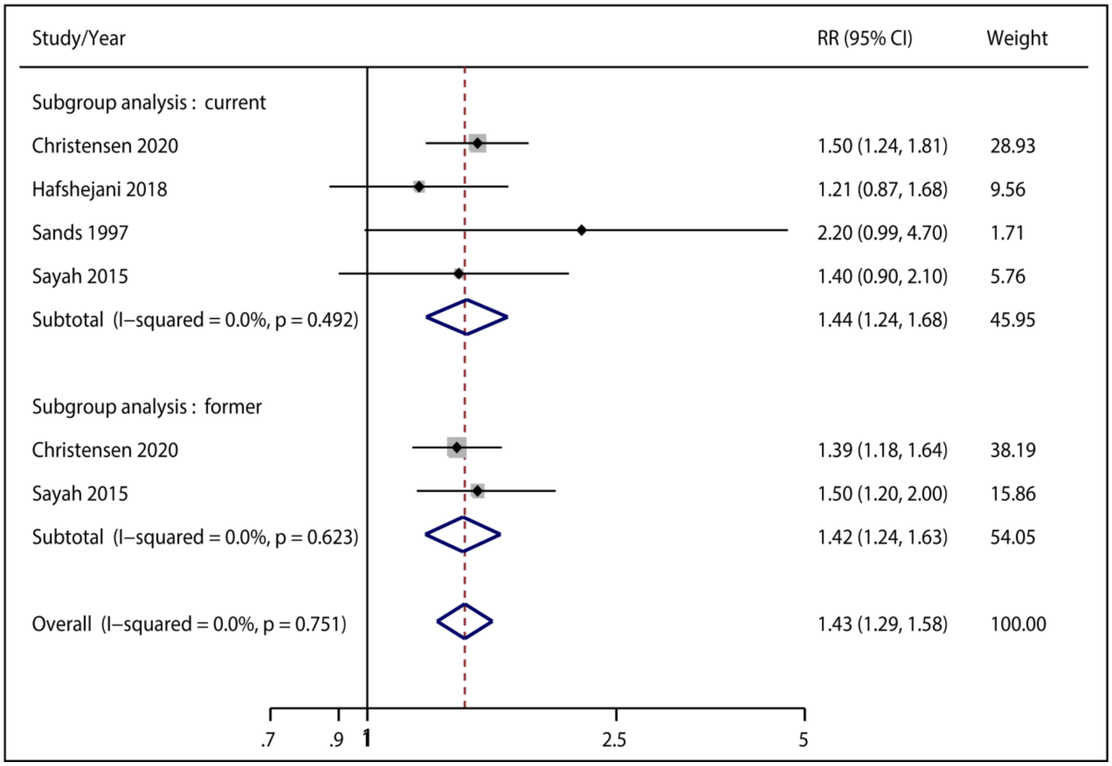

Figure 2- A. Association of smoking with diabetic peripheral neuropathy; B. Subgroup analysis of association of smoking with diabetic peripheral neuropathy.

Supplementary Figure 3. BMI

A

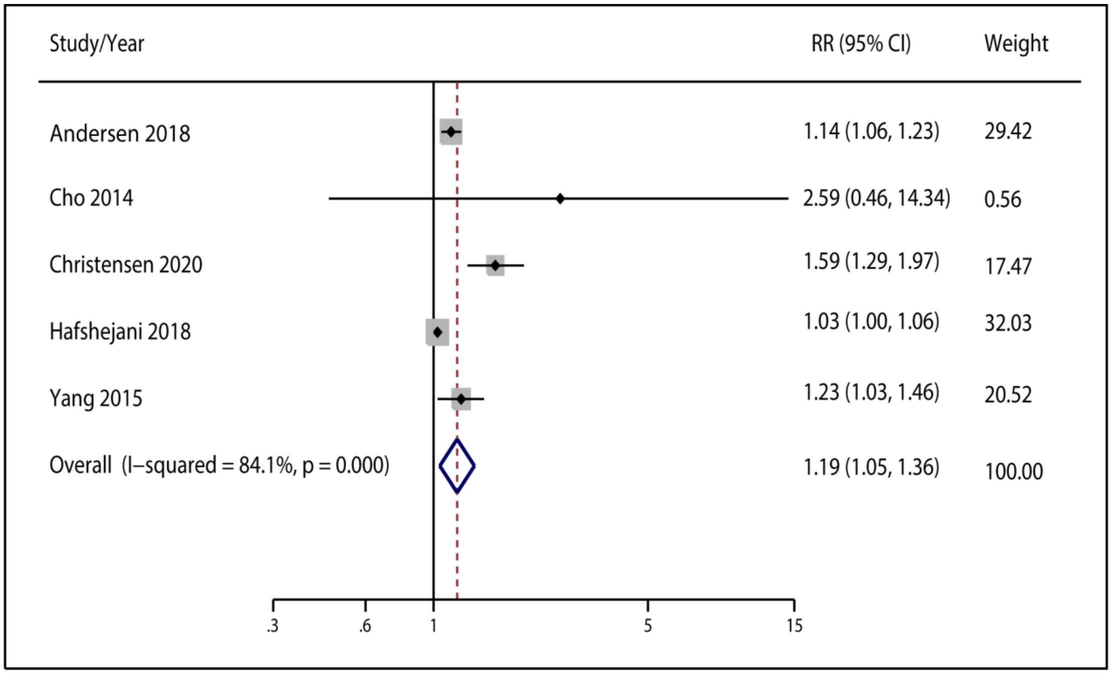

B

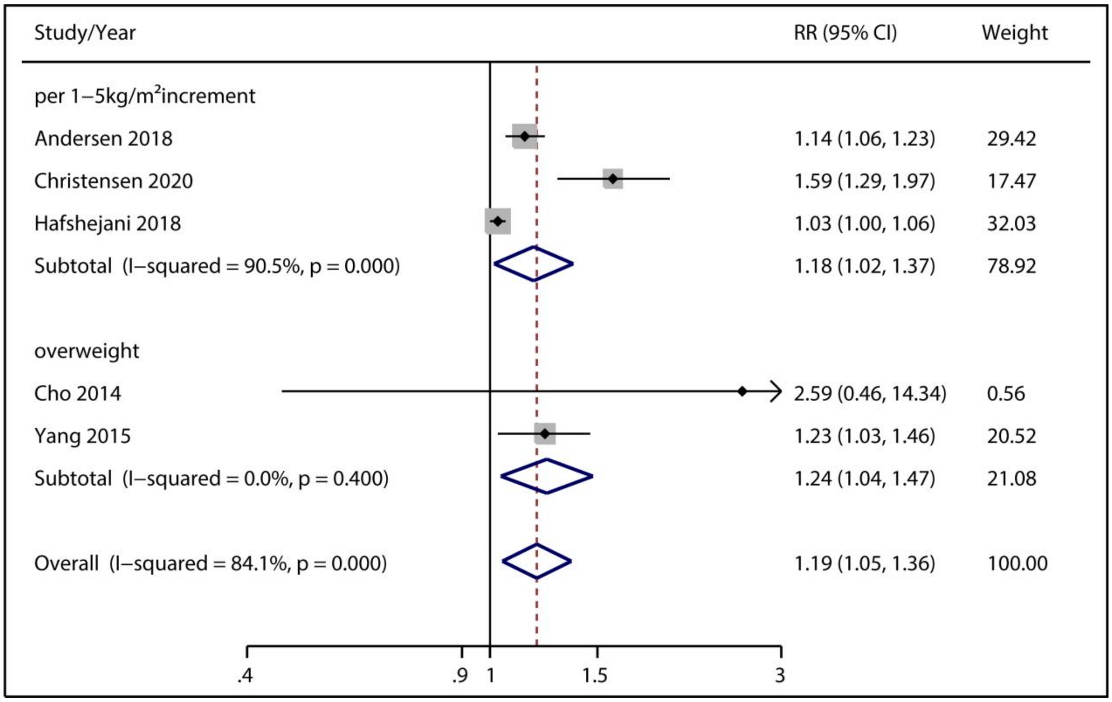

Figure 3- A. Association of BMI with diabetic peripheral neuropathy; B. Subgroup analysis of association of BMI with diabetic peripheral neuropathy.

Supplementary Figure 4. Duration of diabetes

A

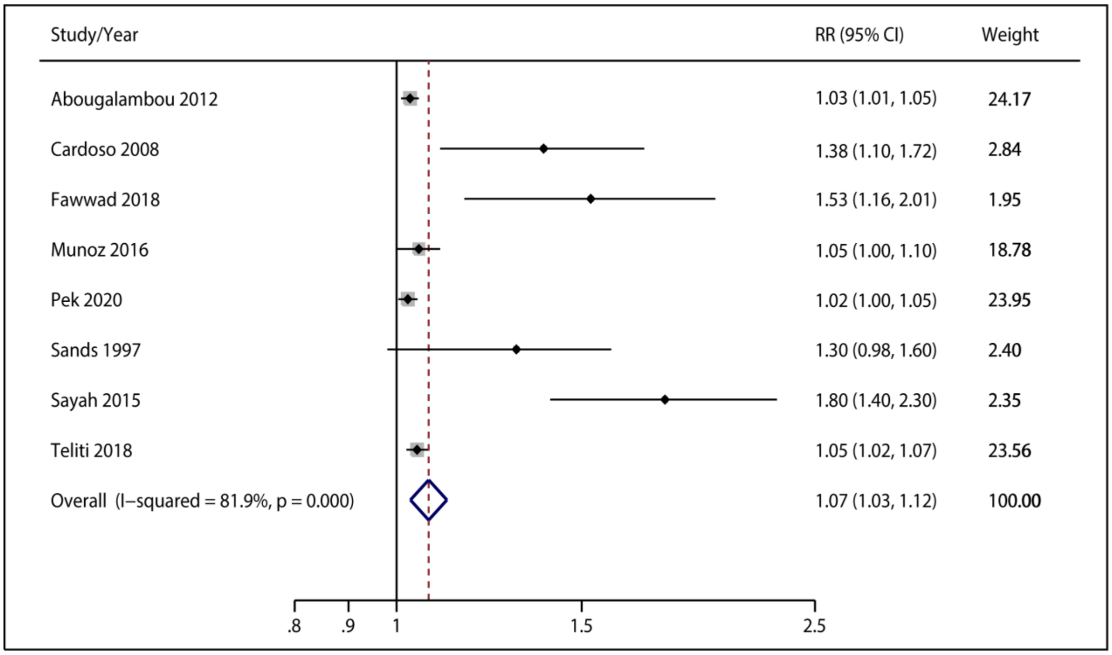

B

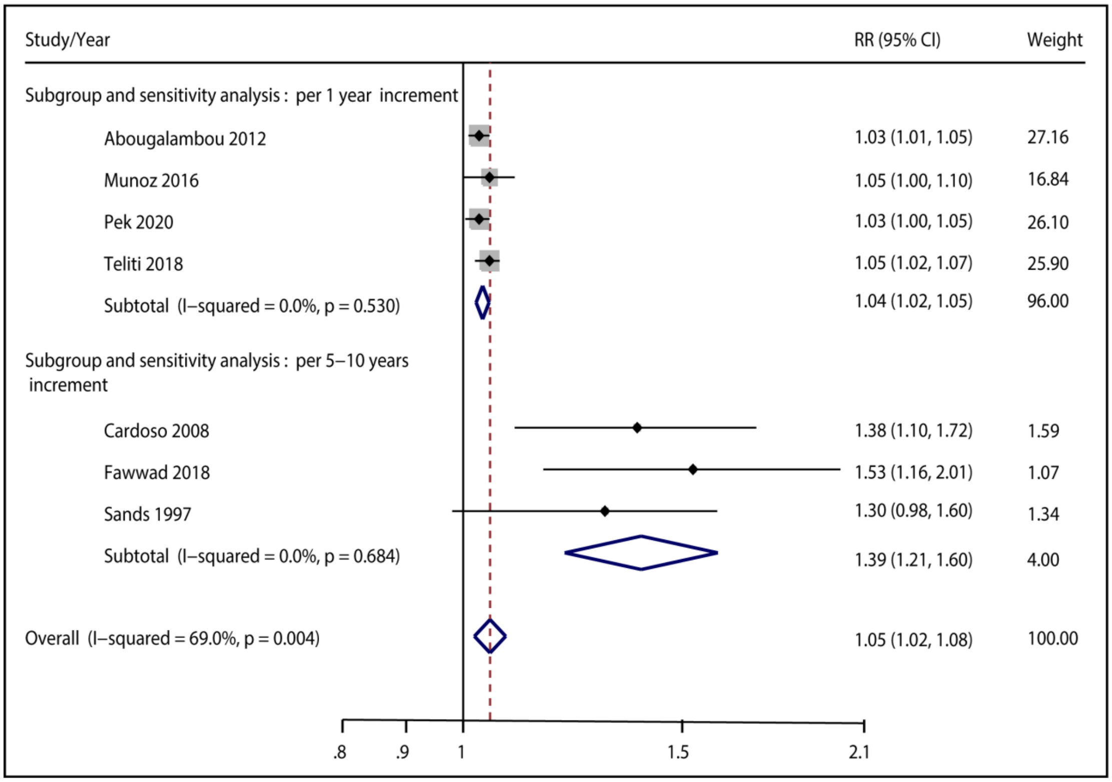

Figure 4- A. Association of duration of diabetes with diabetic peripheral neuropathy; B. Subgroup analysis and sensitivity analysis of association of duration of diabetes with diabetic peripheral neuropathy.

Supplementary Figure 5. HbA1c

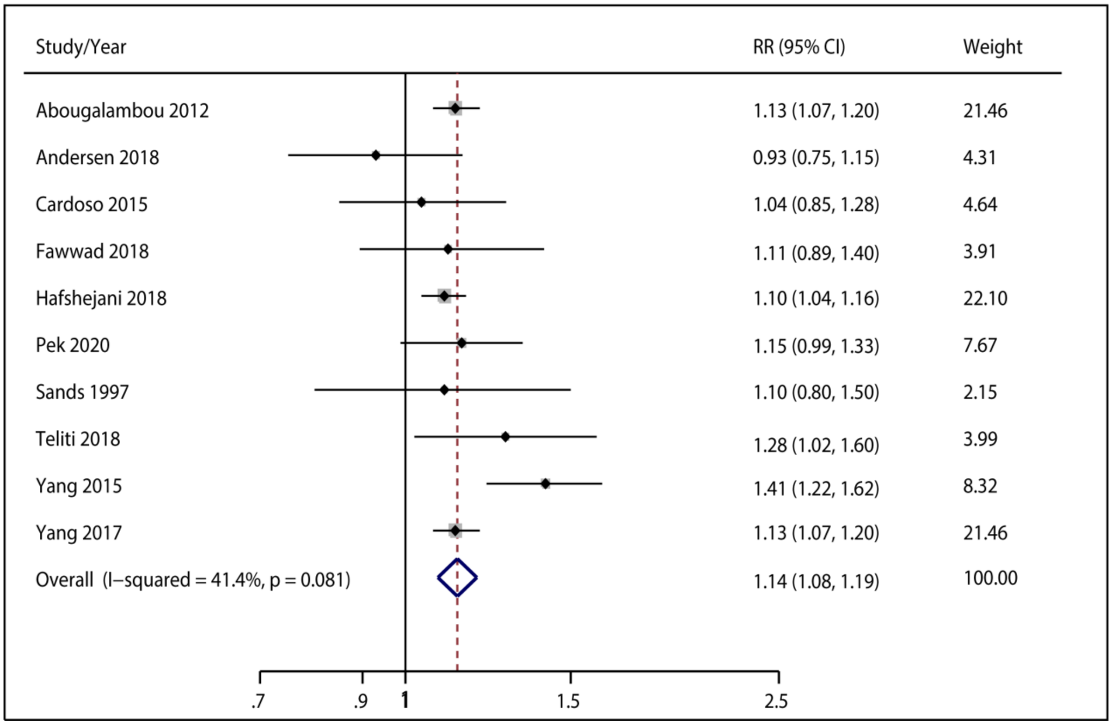

Figure 5. Association of HbA1c with diabetic peripheral neuropathy.

Supplementary Figure 6. Low HDL-c

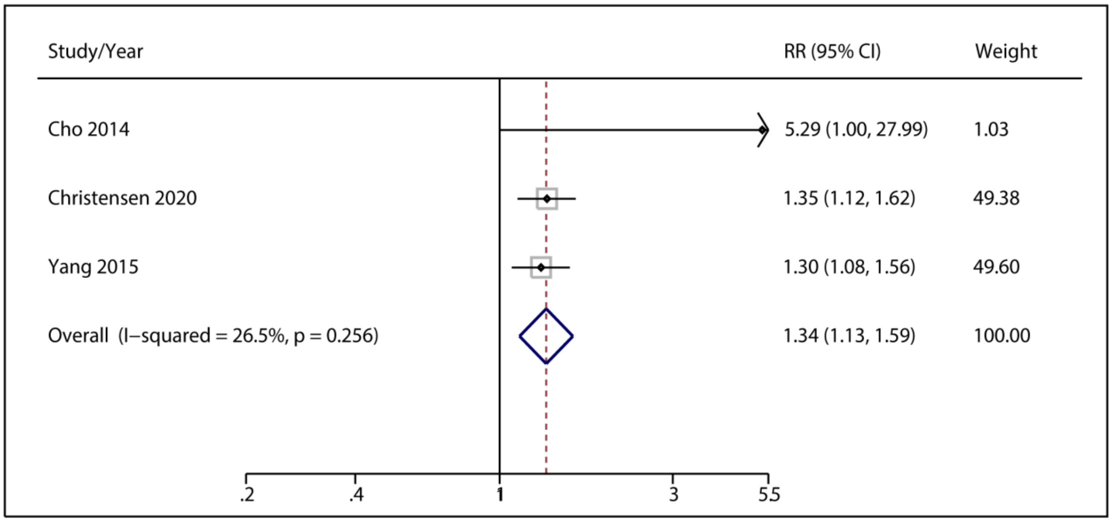

Figure 6. Association of low HDL-c with diabetic peripheral neuropathy.

Supplementary Figure 7. High TG

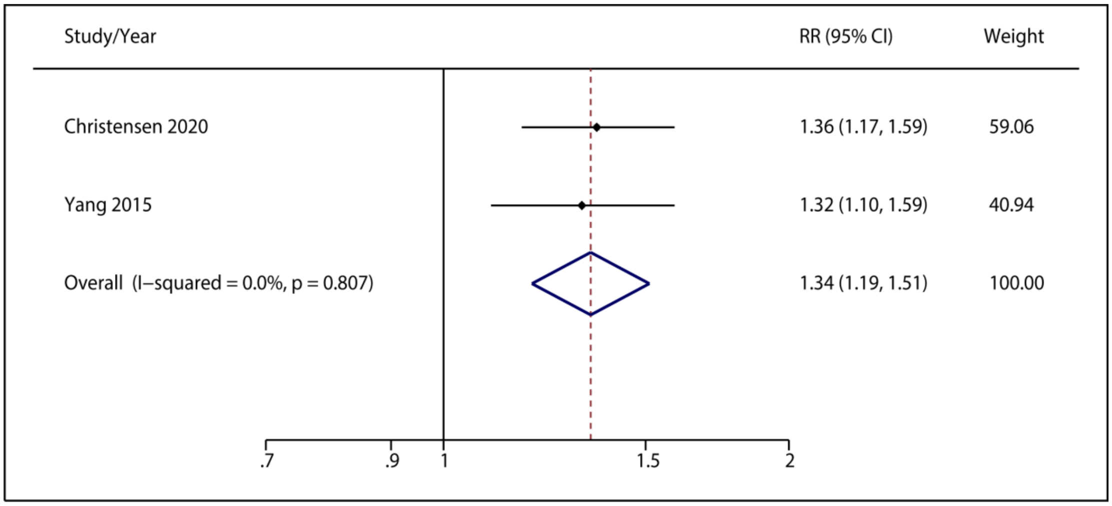

Figure 7. Association of high TG with diabetic peripheral neuropathy.

Supplementary Figure 8. Hypertension

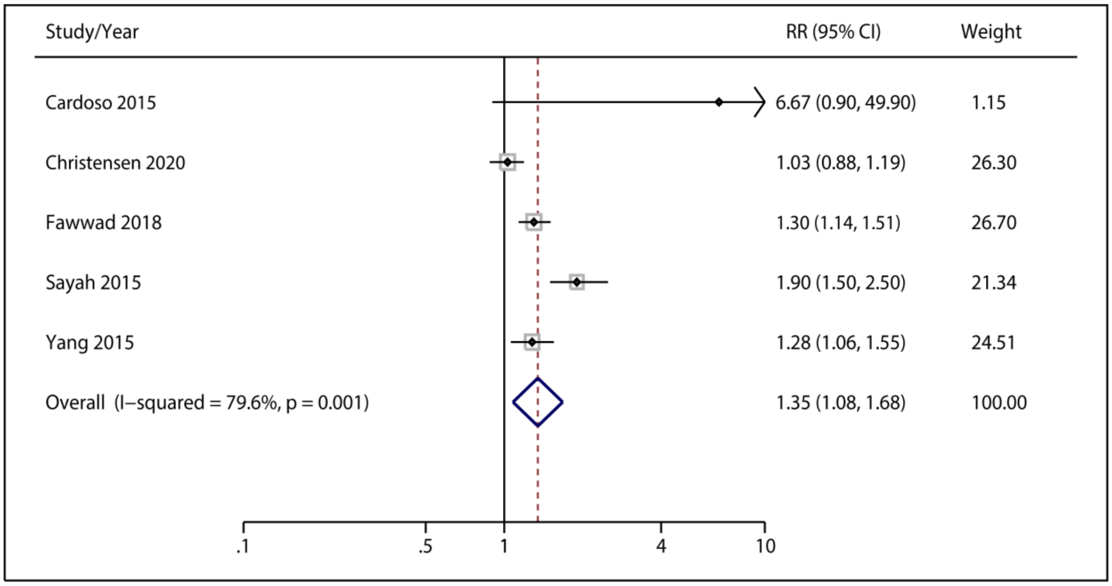

Figure 8. Association of low hypertension with diabetic peripheral neuropathy.

Supplementary Figure 9. Diabetic retinopathy

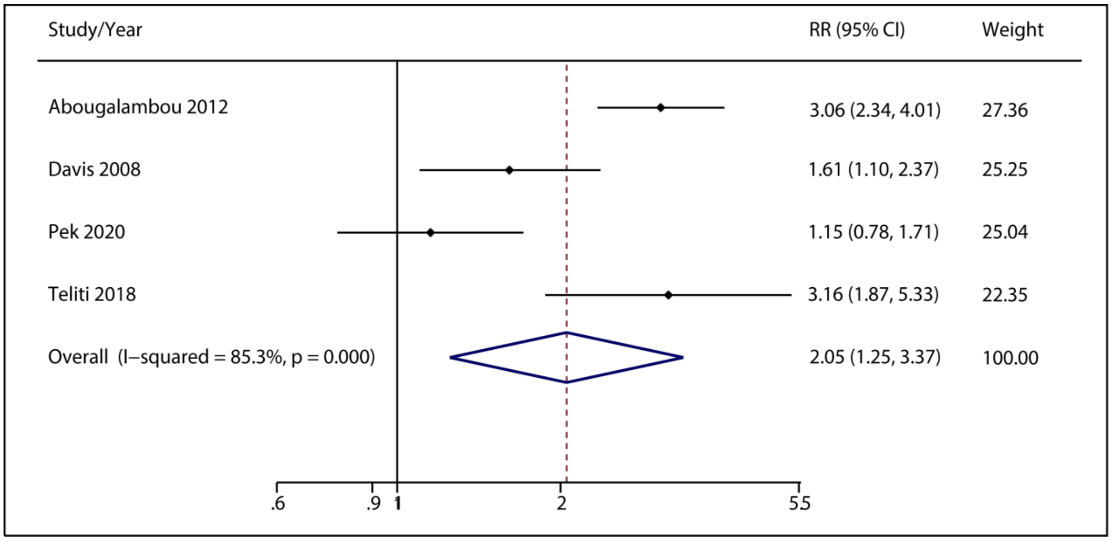

Figure 9. Association of diabetic retinopathy with diabetic peripheral neuropathy.

Supplementary Figure 10. Diabetic kidney disease

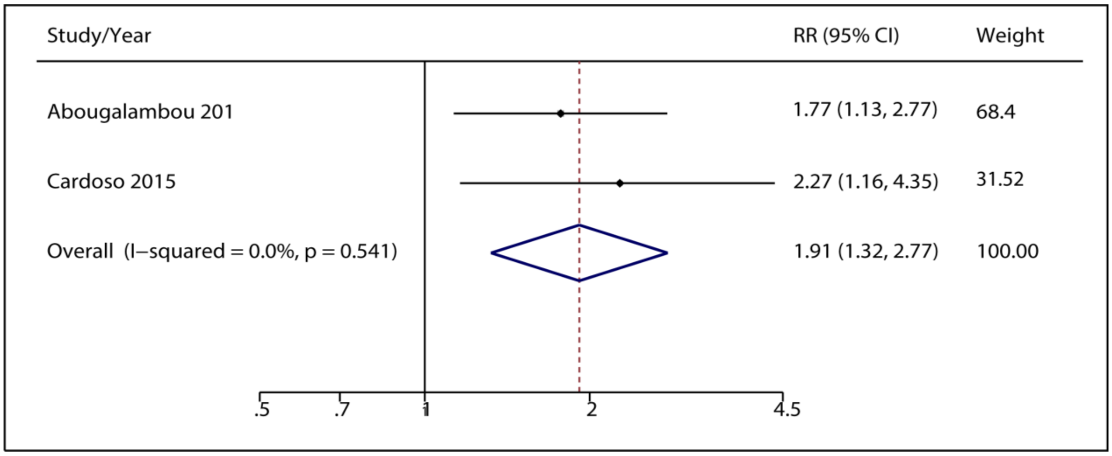

Figure 10. Association of diabetic kidney disease with diabetic peripheral neuropathy.

Supplementary Figure 11. Cardiovascular disease

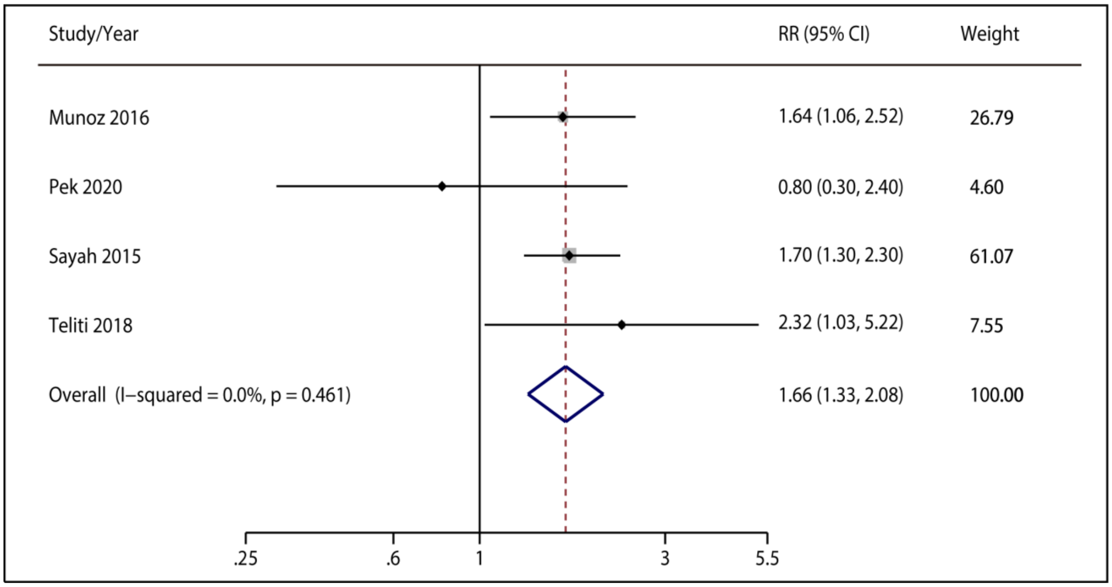

Figure 11. Association of cardiovascular disease with diabetic peripheral neuropathy.

Reference

1. Stroup DF, Berlin JA, Morton SC, Olkin I, Williamson GD, Rennie D, et al. Meta-analysis of observational studies in epidemiology: a proposal for reporting. Meta-analysis Of Observational Studies in Epidemiology (MOOSE) group. Jama. 2000;283(15):2008-12.
2. Moher D, Liberati A, Tetzlaff J, Altman DG. Preferred reporting items for systematic reviews and meta-analyses: the PRISMA statement. BMJ (Clinical research ed). 2009;339:b2535.
3. Tesfaye S, Boulton AJ, Dyck PJ, Freeman R, Horowitz M, Kempler P, et al. Diabetic neuropathies: update on definitions, diagnostic criteria, estimation of severity, and treatments. Diabetes care. 2010;33(10):2285-93.
4. Stang A. Critical evaluation of the Newcastle-Ottawa scale for the assessment of the quality of nonrandomized studies in meta-analyses. Eur J Epidemiol. 2010;25(9):603-5.
5. Pek SLT, Lim SC, Ang K, Kwan PY, Tang WE, Sum CF, et al. Endothelin-1 predicts incident diabetic peripheral neuropathy in Type 2 Diabetes: a cohort study. European journal of endocrinology. 2020;182(4):429-38.
6. Teliti M, Cogni G, Sacchi L, Dagliati A, Marini S, Tibollo V, et al. Risk factors for the development of micro-vascular complications of type 2 diabetes in a single-centre cohort of patients. Diabetes & vascular disease research. 2018;15(5):424-32.
7. Mohammadian-Hafshejani A, Majdzadeh R, Mansournia N, Mansournia MA. Risk factors of peripheral neuropathy in patients with type 2 diabetes in Isfahan: Results of a cohort study in Iran. Biomedical Research and Therapy. 2018;5(12):2926-36.
8. Fawwad A, Mustafa N, Zafar AB, Khalid M. Incidence of microvascular complications of type 2 diabetes: A 12 year longitudinal study from Karachi-Pakistan. Pakistan Journal of Medical Sciences. 2018;34(5):1058-63.
9. Aryan Z, Ghajar A, Faghihi-Kashani S, Afarideh M, Nakhjavani M, Esteghamati A. Baseline High-Sensitivity C-Reactive Protein Predicts Macrovascular and Microvascular Complications of Type 2 Diabetes: A Population-Based Study. Annals of nutrition & metabolism. 2018;72(4):287-95.
10. Andersen ST, Witte DR, Dalsgaard EM, Andersen H, Nawroth P, Fleming T, et al. Risk Factors for Incident Diabetic Polyneuropathy in a Cohort With Screen-Detected Type 2 Diabetes Followed for 13 Years: ADDITION-Denmark. Diabetes care. 2018;41(5):1068-75.
11. Yang CP, Li CI, Liu CS, Lin WY, Hwang KL, Yang SY, et al. Variability of fasting plasma glucose increased risks of diabetic polyneuropathy in T2DM. Neurology. 2017;88(10):944-51.
12. Yang CP, Lin CC, Li CI, Liu CS, Lin WY, Hwang KL, et al. Cardiovascular risk factors increase the risks of diabetic peripheral neuropathy in patients with type 2 diabetes mellitus. Medicine (United States). 2015;94(42):e1783.
13. Cardoso CRL, Moran CBM, Marinho FS, Ferreira MT, Salles GF. Increased aortic stiffness predicts future development and progression of peripheral neuropathy in patients with type 2 diabetes: the Rio de Janeiro Type 2 Diabetes Cohort Study. Diabetologia. 2015;58(9):2161-8.
14. Ybarra-Muñoz J, Jurado-Campos J, Garcia-Gil M, Zabaleta-Del-Olmo E, Mir-Coll T, Zabalegui A, et al. Cardiovascular disease predicts diabetic peripheral polyneuropathy in subjects with type 2 diabetes: A 10-year prospective study. European Journal of Cardiovascular Nursing. 2014;15(4):248-54.
15. Cardoso CR, Salles GF. Predictors of development and progression of microvascular complications in a cohort of Brazilian type 2 diabetic patients. J Diabetes Complications. 2008;22(3):164-70.
16. Abougalambou SSI, Abougalambou AS. Explorative study on diabetes neuropathy among type II diabetic patients in Universiti Sains Malaysia Hospital. Diabetes and Metabolic Syndrome: Clinical Research and Reviews. 2012;6(3):167-72.
17. Davis TME, Yeap BB, Davis WA, Bruce DG. Lipid-lowering therapy and peripheral sensory neuropathy in type 2 diabetes: The Fremantle Diabetes Study. Diabetologia. 2008;51(4):562-6.

18. Sands ML, Shetterly SM, Franklin GM, Hamman RF. Incidence of distal symmetric (sensory) neuropathy in NIDDM. The San Luis Valley Diabetes Study. Diabetes Care. 1997;20(3):322-9.
19. Chuengsamarn S, Rattanamongkolgul S, Sittithumcharee G, Jirawatnotai S. Association of serum high-sensitivity C-reactive protein with metabolic control and diabetic chronic vascular complications in patients with type 2 diabetes. Diabetes & metabolic syndrome. 2017;11(2):103-8.
20. Cho YN, Lee KO, Jeong J, Park HJ, Kim SM, Shin HY, et al. The role of insulin resistance in diabetic neuropathy in Koreans with type 2 diabetes mellitus: A 6-year follow-up study. Yonsei Medical Journal. 2014;55(3):700-8.
21. Christensen DH, Knudsen ST, Gylfadottir SS, Christensen LB, Nielsen JS, Beck-Nielsen H, et al. Metabolic Factors, Lifestyle Habits, and Possible Polyneuropathy in Early Type 2 Diabetes: A Nationwide Study of 5,249 Patients in the Danish Centre for Strategic Research in Type 2 Diabetes (DD2) Cohort. Diabetes care. 2020.
22. Al Sayah F, Soprovich A, Qiu W, Edwards AL, Johnson JA. Diabetic Foot Disease, Self-Care and Clinical Monitoring in Adults with Type 2 Diabetes: The Alberta's Caring for Diabetes (ABCD) Cohort Study. Canadian journal of diabetes. 2015;39 Suppl 3:S120-6.
